# Investigating the Orientation of an Interfacially Adsorbed Monoclonal Antibody and Its Fragments Using Neutron Reflection

**DOI:** 10.1021/acs.molpharmaceut.2c00864

**Published:** 2023-02-16

**Authors:** Sean Ruane, Zongyi Li, Peter Hollowell, Arwel Hughes, Jim Warwicker, John R. P. Webster, Christopher F. van der Walle, Cavan Kalonia, Jian R. Lu

**Affiliations:** † Biological Physics Laboratory, School of Physics and Astronomy, University of Manchester, Oxford Road, Schuster Building, Manchester M13 9PL, U.K.; ‡ ISIS Neutron Facility, STFC, Chilton, Didcot OX11 0QZ, U.K.; § Division of Molecular and Cellular Function, Manchester Institute of Biotechnology, University of Manchester, Oxford Road, Manchester M13 9PL, U.K.; ∥ Dosage Form Design and Development, Biopharmaceutical Development, AstraZeneca, Cambridge CB21 6GH, U.K.; ⊥ Dosage Form Design and Development, AstraZeneca, Gaithersburg, Maryland 20878, United States

**Keywords:** monoclonal antibody, bioengineered antibody, interfacial adsorption, conformational orientation, unfolding, structural deformation, neutron reflection, computer modeling

## Abstract

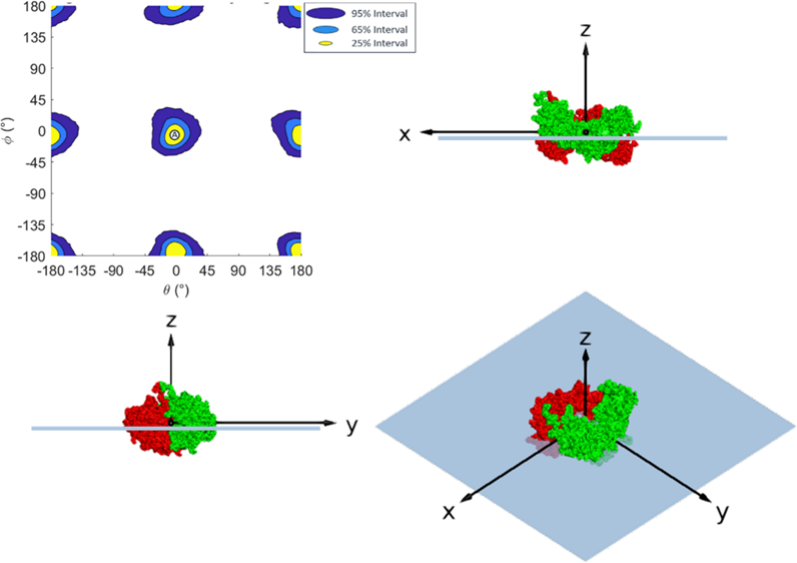

Interfacial adsorption is a molecular process occurring during the production, purification, transport, and storage of antibodies, with a direct impact on their structural stability and subsequent implications on their bioactivities. While the average conformational orientation of an adsorbed protein can be readily determined, its associated structures are more complex to characterize. Neutron reflection has been used in this work to investigate the conformational orientations of the monoclonal antibody COE-3 and its Fab and Fc fragments at the oil/water and air/water interfaces. Rigid body rotation modeling was found to be suitable for globular and relatively rigid proteins such as the Fab and Fc fragments but less so for relatively flexible proteins such as full COE-3. Fab and Fc fragments adopted a ‘flat-on’ orientation at the air/water interface, minimizing the thickness of the protein layer, but they adopted a substantially tilted orientation at the oil/water interface with increased layer thickness. In contrast, COE-3 was found to adsorb in tilted orientations at both interfaces, with one fragment protruding into the solution. This work demonstrates that rigid-body modeling can provide additional insights into protein layers at various interfaces relevant to bioprocess engineering.

## 1 Introduction

Protein adsorption is a phenomenon with implications in the development of biotherapeutic proteins such as monoclonal antibodies (mAbs), where interfacial adsorption occurs during mAb production, filling, transport, storage, and administration. mAbs are fast becoming an important component of the global pharmaceutical market, with more than 50 drugs available in the USA, and the number has doubled over the past 5 years.^1,2^

Therapeutic mAbs are commonly designed by modifying natural human immunoglobulin G (IgG) antibodies via sequence modifications.^3^ These changes can alter the stability of the mAb, increasing its proclivity for aggregation and structural unfolding.^3,4^ IgG-type antibodies consist of 4 polypeptide chains (2 identical ‘light’ chains and 2 identical ‘heavy’ chains), which are linked by disulfide bonds to form two antigen-binding fragments (Fabs) and one crystallization fragment (Fc) connected via a hinge region. mAbs and their fragments, like many other proteins, are amphiphilic due to anisotropic surface distributions of polar, apolar, and charged groups and so will readily adsorb to interfaces.^1,5^ During product filling and storage, mAb drugs will be exposed to a variety of interfaces, such as the glass/water,^6^ steel/water,^7,8^ air/water,^9^ and silicone oil/water^10,11^ interfaces, which have been shown to potentially interfere with production and effectiveness. Exposure to the air/water interface is especially common during manufacturing, transport, and fill/finish processes.^12^ As silicone oil is often used to lubricate the barrel of the syringe, mAb adsorption to the silicone oil/water interface also occurs during storage in pre-filled syringes. Interactions with the air/water^13,14^ and silicone oil/water^10,11,15,16^ interfaces have been implicated in the generation of unwanted particulates, leading to general losses in product efficacy and complication of treatment outcomes.

Various techniques have been explored to characterize protein adsorption, with some of them having also been applied to study mAb adsorption. Neutron reflection (NR) has been used extensively to characterize protein adsorption processes,^17,18^ including several recent studies of adsorption behavior of therapeutic mAbs.^6,7,9,19^ NR is highly sensitive to the adsorbed amount of protein and layer thicknesses, from which information about conformational orientation of the adsorbed mAb molecules can be inferred. Unlike other interface-sensitive techniques such as spectroscopic ellipsometry (SE), NR has inherent sensitivity to structure at Å level as the neutron wavelength used in the measurement is typically 2–30 Å, and substitution of hydrogen with deuterium in water can be used to further enhance structural sensitivity.^20^ Measuring, rather than inferring, the structure at the interface allows more direct investigation of protein behavior at the Å scale.

To unravel how proteins behave at such interfaces, most previous NR work on protein adsorption has used “slab” modeling.^21−23^ In this approach, the adsorbed protein layer is treated as a homogenous cuboid containing various amounts of solvents, proteins, and any other component materials.^24^ This can be extended to multiple layers when necessary, resulting in a better fitting but more complex model with more free parameters that are harder to justify. It is relatively straightforward to apply to complex multi-component systems such as protein-surfactant co-adsorption^25^ and the fitting can be implemented when the exact protein structure is not known or significant structural alteration of the protein has occurred.^26^ Although the slab modeling is simple to implement, it can be difficult to justify its uniqueness when many parameters are involved. When two or more components are involved, it can be difficult to work out meaningful physical implications from such modeling.^27,28^

It is desirable to incorporate the known or predicted secondary and tertiary protein structures into the NR data analysis to explore the protein’s behavior in more detail. Merging NR data analysis with molecular dynamics (MD) simulations will provide a mechanistic understanding of the protein conformational orientation and change.^29−31^ MD techniques, however, are computationally challenging,^32^ especially with high molecular-weight proteins, for example, those commonly found in biopharmaceuticals.^33^ Even with large-scale computational facilities, all or unified-atom simulations of full-length mAbs are slow, especially when aiming at producing simulations on timescales relevant to adsorption processes.^34^ More coarse-grained models, such as modeling individual residues or domains rather than constituent atoms, can provide more computationally tractable solutions.^35−37^ However, coarse graining in a manner that maintains relevance to the real system can be challenging.^33,38,39^ If significant changes from the known structure of the protein are not expected its orientation and penetration into the interface can be modeled.^22,40^ Rigid-body rotation modeling can work as a computationally inexpensive alternative to MD techniques or as an intermediate model to compare to the full MD simulations, while providing more in-depth analysis than slab modeling.^22^ Rigid-body modeling has already been explored by McGillivray et al., who used the crystal structure to localize the α-hemolysin channel protein of*Staphylococcus aureus* in a bilayer lipid membrane.^41^ Nanda et al. applied a similar model to determine the most probable orientation and penetration of the HIV-1 Gag protein into a model viral membrane.^40^

This study aims to demonstrate how rigid-body rotation modeling can give useful information in the context of protein adsorption by comparing the adsorption of a bioengineered mAb COE-3 and its Fab and Fc fragments at the air/water and oil/water interfaces. Investigating the orientation of the adsorbed proteins has the potential to offer more detailed insights into their interfacial behavior. Against the commonly adopted slab layer analysis, rigid-body rotation modeling could help determine the areas of a protein interacting with specific components of the system, for example, the particular residues in contact with the oil or exposed to air or the residues promoting oligomerization. This work could thus offer new insights into the mechanism of surface-mediated incompatibilities and help inform formulation, protein engineering, or process-based mitigation strategies. Rigid-body modeling could also provide a useful starting point for producing more advanced models, combining with volume-occupancy or spline models to investigate how surfactant-protein systems form complex layers^25^ to avoid further protein adsorption.

## 2 Experimental Methods

### 2.1 Materials

COE-3 is a full-length monoclonal antibody of the IgG1 subtype, with a non-glycosylated molecular weight (MW) of approximately 146 kDa. Its Fc has a MW of ∼50 kDa and each of its Fabs has a MW of ∼47 kDa. COE-3 was provided by AstraZeneca at 46.4 mg/mL in 25 mM histidine/histidine hydrochloride buffer (HIS) with 7% w/v sucrose. COE-3 was cleaved into its constituent fragments by digestion with papain and then separated using cation exchange chromatography. The fragments were then exchanged into phosphate buffer and re-concentrated using ultrafiltration and spin filtration to 47.74 mg/mL for Fc and 50.4 mg/mL for Fab. Proteins were directly diluted into measurement buffers of the desired isotopic contrast. All measurements were made in pH 5.5 HIS buffer at an ionic strength of 25 mM and at a temperature of 20 ± 3 °C. The full sequence for COE-3 has been published in previous work.^9^

### 2.2 Neutron Reflection

NR has been used extensively as a depth-profiling technique for exploring the conformational orientation of proteins and other molecules at interfaces.^21^ Specular NR provides excellent sensitivity to the structure at the scale of individual protein monolayers in the direction normal to the interface, allowing investigation of protein adsorption at the monolayer level. Neutron scattering techniques, such as NR, are primarily sensitive to changes in scattering length density (SLD), the sum of neutron scattering lengths in a material per unit volume.

Unlike techniques such as X-ray reflection which are sensitive to soft matter systems on similar scales, NR can provide increased structural sensitivity via contrast variation.^20^ As hydrogen (^1^H) and deuterium (^2^H) have very different neutron scattering lengths (−3.74 × 10^–5^ Å for hydrogen and 6.67 × 10^–5^ Å for deuterium), substituting hydrogen for deuterium in the material or solvent allows two or more neutron measurements from the same system under different contrasts, thus reducing the number of potential solutions dramatically. For example, null reflecting water (NRW) with SLD = 0 matches that of air, with no specular signal at the air/NRW water interface, leaving the adsorbed protein and its associated ligands as the only reflecting component. Parallel measurement could be made by contrast matching the aqueous solution to the protein (CM Protein, SLD = 2.56 × 10^–6^ Å^–2^ for COE-3), leaving the protein region that is exposed to air as the sensitive region to be determined.

Measurements of COE-3 adsorption at the air/water interface were taken at 50 ppm in 4 isotopic contrasts; NRW, CM Protein, SLD = 4.5 × 10^–6^ Å^–2^ (CM 4.5), and D_2_O (SLD = 6.35 × 10^–6^ Å^–2^). Measurements of COE-3 adsorption at the oil/water interface were taken at 50 pm and 200 ppm in 3 isotopic contrasts: H_2_O (SLD = −0.56 × 10^–6^ Å^–2^), CM protein, and contrast matched to the sapphire substrate (CM Sapphire, SLD = 5.65 × 10^–6^ Å^–2^). All measurements were made in pH 5.5 HIS buffer. For oil/water measurements, n-hexadecane oil was used as the model oil because of the availability of the deuterated oil. A 1.4 ± 0.1 μm hexadecane film was formed using the spin-freeze–thaw method by Zarbakhsh et al.,^42^ The SLD of the oil was contrast matched to the sapphire substrate, minimizing the reflection at the solid/oil interface as described previously.^19^ The experimental data used in this study have been previously analyzed using slab modeling, with the data obtained from the oil/water interface by Ruane et al.^19^ and the data from the air/water interface by Li et al.^9^ The oil/water reflectivity measurements were taken at a single incidence angle only due to experimental limitations,^44^ with the overall *Q*-range of 0.018–0.2 Å^–1^. In contrast, data in the *Q*-range of 0.012–0.4 Å^–1^ for the neutron reflectivity profiles were measured at the air/water interface. These studies have examined how the adsorbed amount varied with pH and COE-3 concentration, highlighting how NR measurements under different contrasts could improve sensitivity and resolution. In addition to the previous data, new measurements of adsorption of Fab and Fc at 50 ppm at the air/water interface under the isotopic contrast of CM 4.5 were also taken on the FIGARO reflectometer at Institut Laue-Langevin at Grenoble, France,^43^ under the same solution conditions (pH 5.5, His buffer). These air/water reflectivity measurements were taken over the *Q*-range of 0.0063–0.32 Å^–1^.

### 2.3 Homology Modeling

Homology models of COE-3 and its Fab and Fc fragments were taken from a previous study by Singh et al., comparing electrostatic parameters calculated from models to those obtained experimentally in light scattering experiments.^45^ To prepare for rigid-body modeling, the models were energy minimized for 50,000 steps and then relaxed in NVT (constant particle number, volume, temperature for 500,000 steps of 1 ns step time) and NPT (constant number, pressure, temperature for 5,000,000 steps, 10 ns step time) ensembles using GROMACS, using the GROMOS96 43a1 force field at 293 K. Because of the globular stability of the protein and a small movement of the atoms relative to the scales at which NR is sensitive, little difference was observed between the original models and equilibrated models in subsequent handling.

### 2.4 Rigid-Body Rotation Modeling (RBRM)

Multiple formalisms can be used to express 3D rotations. Euler angle rotation, in which rotations are performed around the cartesian angle axes, is a relatively intuitive system.^46^ Three total rotations involving at least 2 axes are needed to describe all possible 3D rotations. However, as NR is only sensitive to the SLD profile along the *z*-axis (normal to the interface), rotations around this axis do not affect the results. Thus, only 2 angles are needed to describe the full orientation as determined by NR, reducing the number of necessary parameters.

In the Euler angle formalism, rotations are not commutative, so a rotation around the *x*-axis followed by a rotation around the *y*-axis (denoted as an *XY* rotation) does not result in the same final state as if the order of rotations is reversed. There are 12 possible sets of 3-angle rotation formalisms (e.g. *XYZ*, *XZX*, etc.) all of which can access the full 3D rotation space with 3 angles and 8 of which can access the *Z*-invariant rotation space with just 2 angles. The NR rotation modeling work by Nanda et al.^40^ used a *ZY* rotation system, in which the protein was first rotated by angle θ around the *z*-axis and then rotated by angle ϕ around the *y*-axis. In this work, the primary mode of rotation is instead the *XY* system, in which the protein is rotated by θ around the *x*-axis and then by ϕ around the *y*-axis. This system provides a more intuitive understanding of the results that were found in this analysis, though both rotation systems can be useful for demonstrating different symmetries in results.

For interfacial adsorption processes at liquid–liquid and liquid-air interfaces, the penetration of the protein from solution onto the interface is an important consideration when investigating protein behavior and has a large impact on the model. Penetration was modeled by including a penetration depth parameter in the model, corresponding to the distance in *z* the protein protruded from the solution/substrate interface into the substrate phase. Thus, a penetration depth of 0 corresponds to the interface being located at the minimum *z* value of the protein, with the protein immersed entirely in the solution.

**Figure 1:**
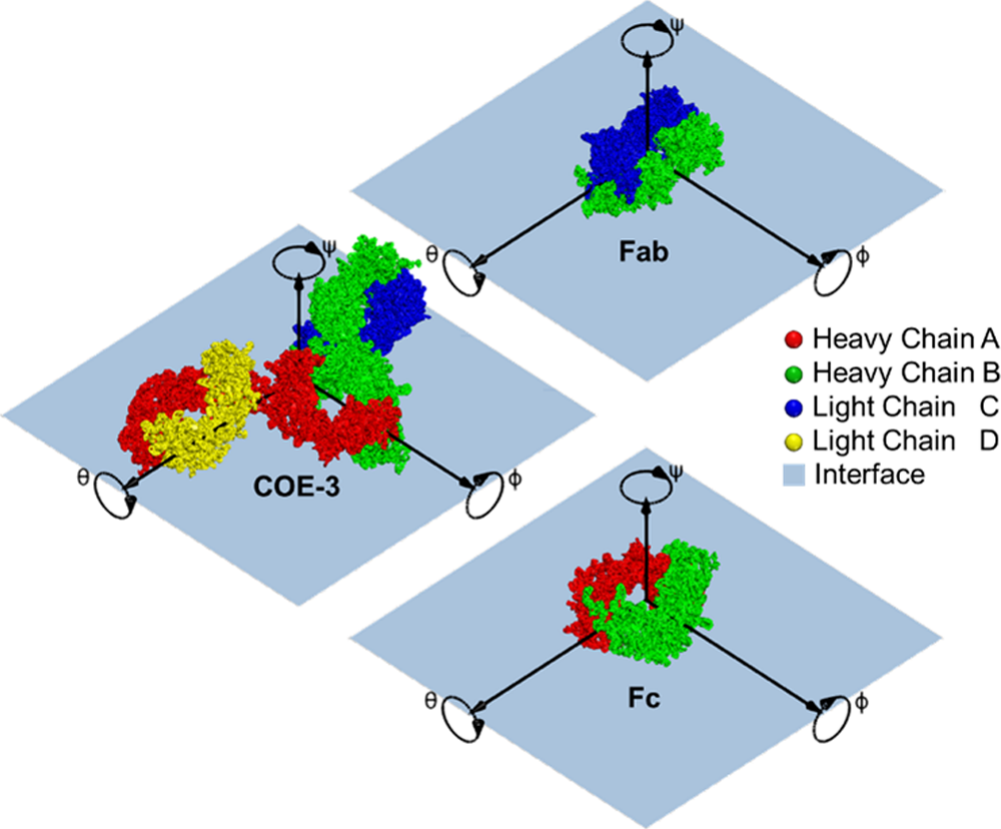
Initial orientation for COE-3 and its Fab and Fc fragments. Note the color-coding scheme throughout: red and green are heavy chains, and blue and yellow are light chains. Visualizations are taken with the aqueous solution layer above and substrate (air/oil) layer below. Visualizations were created in PyMol.^47^

For the initial protein orientation (corresponding to θ = 0, ϕ = 0), the proteins were centered around the origin with their major axis aligned along the *y*-axis and semi-major axis along the *x*-axis. Figure 1 shows the initial orientations and rotational axes for COE-3, Fab and Fc, respectively, under RBRM. This figure and all subsequent figures are shown with the aqueous layer on top and the air or oil substrate on the bottom. This is inverted from the geometry of the actual experiment in which the oil or air substrate is above the solution, but as the majority of protein remains in the solution layer, this orientation allows for clearer observation of the adsorbed conformation.

**Figure 2:**
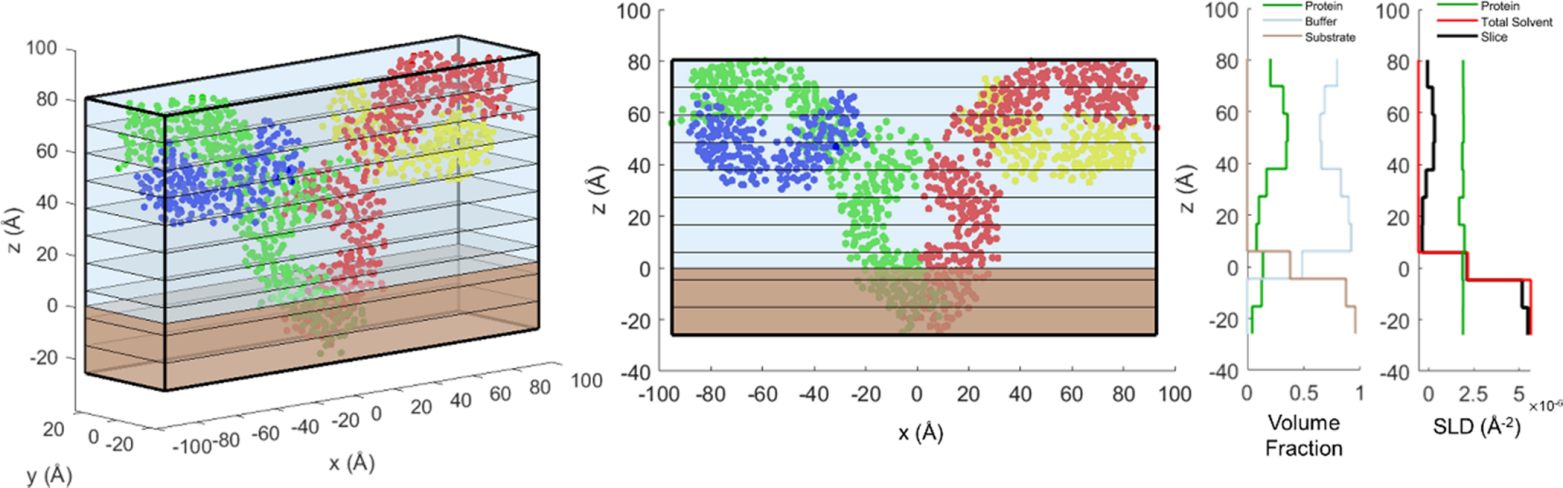
Illustrations of how the box around the protein was constructed with 3D view (left), front view (middle), and split into slices and the resulting volume fractions and SLD profiles (right) for each slice and its components. In the figure, only 10 indicative slices were used to improve legibility; in the actual model, 50 slices were used. Note that the brown region represents oil or air.

The two Euler angles can be used to construct a rotation matrix;^46^ multiplying the position of each residue in the structure by the matrix will produce the desired rotation. After the structure is rotated, the resulting SLD profile can be calculated. As shown in Figure 2, a box is constructed around the rotated protein, with dimensions from minimum extent in *x*, *y*, and *z* to the maximum. This box is then divided along *z* into *n* slices of equal thickness *d*. Within each slice, the volume of protein in the slice *V*_slice_^prot^ and the SLD of the protein in the slice SLD_slice_^prot^ can then be found by the equation:
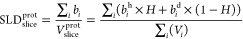
1where *b_i_^h^* and *b_i_^d^* are the scattering lengths of residue *i* in H_2_O and D_2_O respectively, *V_i_* is the empirically measured volume of residue *i*,^48^ and *H* is the fraction of H_2_O in the aqueous solvent by volume. From these values, the total SLD of the slice can be found:
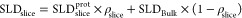
2where ρ_slice_ is the fraction of protein in the slice by volume and SLD_Bulk_ is the SLD of the aqueous solution (SLD_Solvent_) if the slice is below the penetration length and if the SLD of the substrate (SLD_Substrate_), that is oil or air, is above the penetration length. For the slice in which the penetration distance falls, that is, the slice which is cut through at the interface, SLD_Bulk_ is given by:
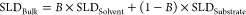
3where *B* is the relative fraction of aqueous solvent to substrate bulk in the slice:
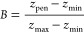
4where *z*_min_ and *z*_max_ are the lower and upper *z* bounds of the slice, respectively, and *z*_pen_ is the penetration depth. This method was adopted as it is computationally simpler than dividing the slice into two, while maintaining consistent slice widths and providing very similar results (unless excessively large slices are used).

Once this is achieved for each slice, the total volume fraction of proteins in the box can be found:
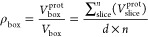
5

However, this is insufficient to calculate the hydration of the layer; as the box has an arbitrary boundary around the protein, the volume of the protein in the box may not be the same as that of the layer. One can imagine that the protein boxes could be spaced further apart in a loosely packed layer or the boxes could overlap in a densely packed layer, especially in the case of non-spherical proteins such as COE-3. Thus, a normalizing hydration parameter ρ_norm_ is required, to relate the box hydration and the desired final hydration:
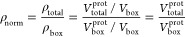
6where ρ_total_ is the actual final volume fraction of protein in a box of dimension *V*_box_. Thus, ρ_norm_ can be less than one, representing greater hydration than ρ_box_, or greater than one, representing proteins being more densely packed. For each slice:
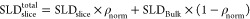
7where SLD_Bulk_ is as in eq 3.ρ_total_ can be used directly as a fitting parameter, but as ρ_box_ is liable to change significantly with orientation, it is not overly useful as a comparative variable. Instead, ρ_total_ can be calculated from Γ, the adsorbed mass per unit area:
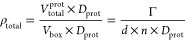
8where *D*_prot_ is the density of the protein, calculated separately for each protein using Fischer et al.’s MW dependent formula.^49^ Due to the high sensitivity of NR to Γ, this provides a much more stable variable. This process results in an ensemble of slices corresponding to the protein’s SLD profile, from which reflectivity data can then be simulated using the Born & Wolf optical matrix method.^50,51^ The rotation model was developed using RasCAL’s custom model capabilities.^52^ For practicality and computational simplicity, the model makes some assumptions. First, the volume and SLD of each amino-acid residue are binned within a single slice, while, in reality, the atoms within the residue may be spread across multiple slices. While slice width was not found to have a significant effect within this study, the drawback of this treatment may be very apparent with smaller proteins. More complex models, for example, calculating SLD and volume by individual atom locations, would greatly increase computational workload and complexity. Second, the labile hydrogens within residues were fully exchanged. This may not be true for residues deep within the core of a protein, but the impact of incomplete exchanges on SLD would be well within 10%. Third, this model simulated a monolayer with a single protein orientation, while a range of conformations might actually be adopted. Finally, the protein remained static and rigid. Future work could look into these issues to improve the model.

The rotated structure of the protein can be used to estimate the footprint area per molecule (APM) for a particular orientation, with the amount of the interfacial surface being taken up by a single protein. A simple estimate can be found by determining the convex hull of the molecule in the *X* and *Y* plane, the smallest convex polygon that can fit around all the residues in the plane of the interface.^53,54^ For non-spherical molecules such as COE-3, this may result in a large overestimate due to the concave shape of the molecule. For such molecules, a concave estimate of the boundary area may be a more accurate representation,^55^ with demonstrations of these calculations being shown in Figure S1. Figure S2 shows the calculated footprint APM for the Fab as a function of rotation in θ and ϕ, while Figures S3 and S4 show the same for Fc and the full-length COE-3, respectively. This footprint area can then be compared with the APM measured in the real system from the adsorbed mass, given by the equation:
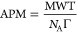
9where MWT is the molecular weight of the molecule and *N*_A_ is Avogadro’s constant. Table S1 lists the calculated footprint and measured APM for each measurement, and these results are discussed further in Section 3.4.

### 2.5 Markov Chain Monte Carlo Analysis

Using a typical minimizing algorithm such as the simplex algorithm^56^ to find a “best fit” orientation, penetration and Γ that minimizes χ^2^ can be a useful analysis technique but gives no information about other potential solutions or confidence in the fitted parameters. Calculating how the χ^2^ value varies with orientation would give qualitative information about which orientations better fit the data but cannot easily be compared in terms of confidence. To solve this issue, Bayesian analysis techniques such as Markov Chain Monte Carlo (MCMC) analysis are useful for investigating the probability distribution of the parameters.

In brief, MCMC uses an iterative process to explore the parameter space such that each step samples the posterior probability distribution of the parameters.^57^ With a large chain, the distribution of the chain steps should approximate the posterior probability distribution of the parameters and so the density of the resulting sample points provides an estimate of the probability density function (PDF) for the parameters. Thus, by analyzing the distribution of the accepted θ values against ϕ values, the probability of the orientation can be investigated, with regions of high probability corresponding to regions of high likelihood, that is, the best fitting regions. The PDF can be estimated by binning the points into a histogram or calculating the kernel density estimate (KDE). A bin width of 5 degrees was used for the histograms, while KDEs were calculated using an adaptive bandwidth diffusion technique.^58^ Credible intervals for orientation were then found by calculating the highest posterior density regions (HPDR) of the aforementioned KDE, the smallest bounded area which contains the desired probability, for example, 65% of the probability volume. As Γ and penetration are univariate parameters distributed roughly in a Gaussian, the confidence intervals were calculated by taking the standard deviation.

Bayesian probability analysis methods use a prior distribution, which represents what is believed to be the most likely distribution of the parameters. In this study, MCMC analyses were performed using a uniform prior, to avoid making strong assumptions about the distribution of the orientation, which were likely to be non-Gaussian. A delayed rejection adaptive metropolis^59,60^ algorithm was used to improve chain convergence and exploration of the parameter space. Due to the rotational symmetry of the problem, the algorithm was modified to include periodic boundary conditions for θ and ϕ, allowing the Markov Chain to wrap around the opposite limit; for example. a step of θ = 3° could travel from θ = – 179 to θ = + 178. This change greatly improves chain mixing and reduces autocorrelation in the results. For models of the Fab and Fc fragments, each MCMC simulation was run for 5 repeats of 200,0000 steps with 200,000 burn-in steps, while for COE-3 due to the high proportion of rejected steps, each MCMC simulation was run for 5 repeats of 16,000,000 steps with 400,000 burn-in steps. Figure S5 shows the MCMC traces for the chains, while Figure S6 shows plots demonstrating the autocorrelation of the same parameters. Figure S7 shows the traces and autocorrelation for the measurement of 50 ppm Fc adsorbed at the air/water interface, shown separately for reasons discussed below in Section 3.2 and the Supporting Information.

While NR data is fitted to a single protein orientation in this work, it is of course possible that there are multiple preferred orientations. With sufficient neutron contrasts involving deuterated proteins or with intelligent experimental design, it is possible to distinguish such states. However, due to the vastly increased complexity of such a model, this is beyond the scope of this study.

## 3 Results and Discussion

Figure S8 shows the measured reflectivity data together with the best fits from the RBRM and the resulting SLD profiles for the adsorption of Fab, Fc, and the whole mAb COE-3 at the air/water and oil/water interfaces. Under each combined interface and solution condition, three isotopic contrasts were applied to highlight the adsorbed protein layer differently. The SLD profiles were obtained from simultaneous fitting to the reflectivity profiles under the three contrasts. The shaded areas shown in Figure S8 mark the 65% confidence intervals of the model fits. For each condition, confidence intervals were determined by taking a sample of 3000 chain steps and calculating the resulting set of SLD profiles and simulated reflectivity data. A 2D interpolation of the set of results was created to find the 65th percentile results for each *Q_z_* value for the reflectivity data and each *z* value for the simulated SLD profile, with further details given in Section 1 of the Supporting Information.

Table S2 shows the previously fitted layer models for each NR measurement^9,19^ and their χ^2^ values compared to the best-fitting orientations, adsorbed amount Γ, and penetration from the rigid-body rotation model with a differential evolution minimizing algorithm.^61^ It can be seen that the best-fit rigid-body model produces significantly lower χ^2^ values than the slab models, suggesting that it produces more accurate SLD profiles. The improvement in χ^2^ is more pronounced when there is an increased signal-noise ratio (more contrasts and increased protein at the interface), especially from the measurements at the air/water interface, producing more accurate SLD profiles. It can thus be assumed that the structures obtained are reasonably reliable and that no large-scale structural changes such as protein deformation or unfolding have occurred.

### 3.1 Fab Adsorbed at Air/Water and Oil/Water Interfaces

Table S3 shows the average penetration for each protein and interface from the rigid body model and also from previously published slab models. As a single value, this penetration appears much higher for both interfaces from the rigid-body models than from the previous slab models. This model-dependent difference is however misleading. Intuitively, the displaced volume due to a sphere penetrating across a boundary will be lower than that of a cube of the same volume penetrating flat-on for the same distance. This effect is amplified by the fact that protein structure files often have protruding residues stretching out as rigid segments. As penetration is measured from the outermost residue, a large penetration distance can correspond to a relatively low penetrated volume. Thus, Table S3 also includes *P*_f_, the fraction of the volume (estimated as the proportion of residues that pass the boundary) mixed with the oil or air substrate. Subplot (b) of Figures S9–S18 in the Supporting Information shows how the *P*_f_ value changes with the orientation of the molecule for each protein at each interface and concentration.

**Figure 3:**
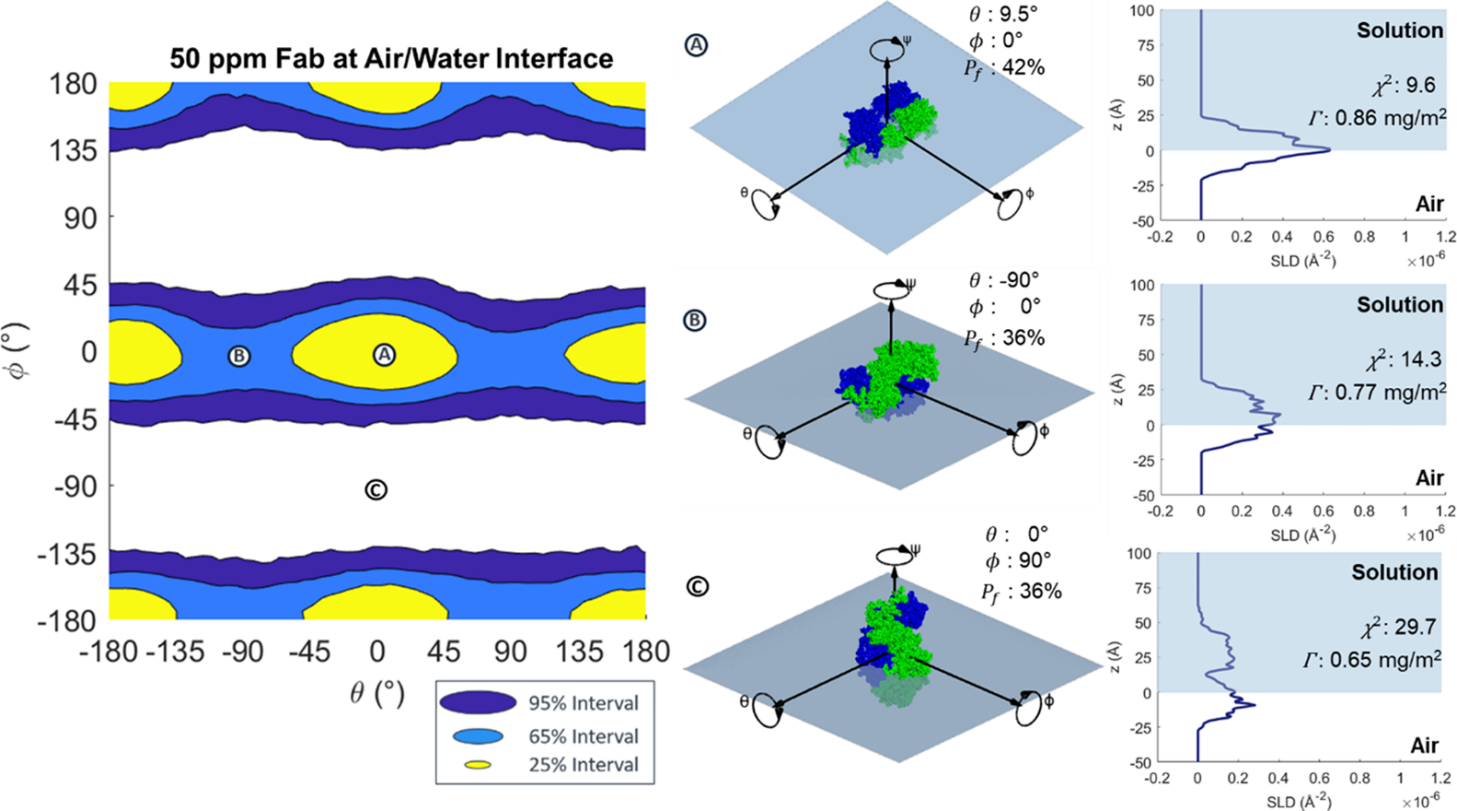
HPDI variation for 50 ppm Fab adsorbed at the air/water interface by plotting change in ϕ versus θ (left), with visualizations (middle) and SLD profiles (right) from key points labeled in the figure. SLD profiles are given in the substrate-matched aqueous contrast, in this case NRW (SLD = 0 × 10^–6^ Å^–2^), to isolate the contribution from the protein.

Figure 3 shows the highest probability density intervals (HPDI) for 50 ppm Fab adsorbed at the air/water interface. Coordinates on the graphs correspond to rotations from (θ = 0°, ϕ = 0°) as displayed in Figure 1. The shaded regions on the graphs correspond to the smallest regions in which a certain probability of the system resides in, roughly equivalent to the smallest areas in which a certain proportion of the accepted MCMC steps is found; for example, ∼25% of the MCMC steps are found within 25% HPDI. As expected from a rotationally symmetric system, the intervals “wrap around” in both θ and ϕ. The supporting figures are shown in Figure S9, with subfigure (a) showing a plot of all the points in the accepted chain, subfigure (b) showing the averaged *P*_f_ value, subfigure (c) showing the KDE from which the HPDI regions were calculated, and subfigure (d) showing a 2D histogram binning the accepted chain in θ vs ϕ, for comparison to KDE. Supporting figures shown for every measurement in this study from Figures S9–S18, with subfigure labels are corresponding to the ones given here.

The most probable regions, the 25% HPDIs, which correspond to regions that best fit the neutron reflectivity data, are centered around (θ = 9.5°, ϕ = 0°) and the regions 180° away in θ and ϕ, which are split by the edges of the boundary. An example of an orientation included in the high-probability regions is the point at (θ = 9.5°, ϕ = 0°), labeled point Ⓐ in Figure 3, which is the best-fitting orientation for Fab at the air/water interface. The corresponding SLD profile follows a similar style, as it is mostly symmetrical along its minor axis and so flipping the protein by 180° will result in a similar SLD profile. This is true for both Fab and Fc fragments and for the full COE-3 mAb.

Here, the best-fitted set of parameters corresponds to the Fab lying approximately ‘flat-on’, with its smallest axis perpendicular to the interface. The thickness of the layer is effectively minimized to ∼50 Å with a high protein monolayer density, as demonstrated at point Ⓐ in Figure 3. Comparing points Ⓐ and Ⓑ in Figure 3 demonstrates that rotating in θ, around the Fab’s long axis, results in a relatively small change in the SLD profile. The Fab tertiary structure approximates a cylinder, so rotating around the axis can only broaden the SLD profile to ∼55 Å. This effect can be seen in relative uniformity in θ of the HPDI plots and supporting posterior plots. Point Ⓒ in Figure 3 demonstrates that rotating around ϕ changes the SLD profile far more significantly, resulting in a much thicker layer. Thus, the Fab is not lying end-on against the interface but rather flat-on.

Previous analysis of Fab adsorption at the air/water interface^9^ showed that the Fab is adsorbed in a dominant flat-on orientation with a 15 ± 2 Å layer above the interface and a 25 ± 2 Å layer below it, with a total thickness around 50 Å corresponding to *P*_f_ = 30 ± 3%. The overall layer thickness is consistent with the rigid-body rotation model used here with a total Fab envelope of 45–60 Å, but the RBRM model reveals a much higher protein density toward its center. The penetration is lower in the slab model, as shown in Table S2, and the rigid body model shows an increased *P*_f_ = 40 ± 10%. These differences arise from model-dependent features. While the slab model adopts an averaged but sharp interfacial transition, the rigid body model retains the shape of the outermost interfacial boundary, resulting in higher penetration and *P*_f_. There is a consistently lower Γ value from the rigid body model across all the concentrations studied at the air/water interface, again pointing to possible model dependent discrepancy.

**Figure 4:**
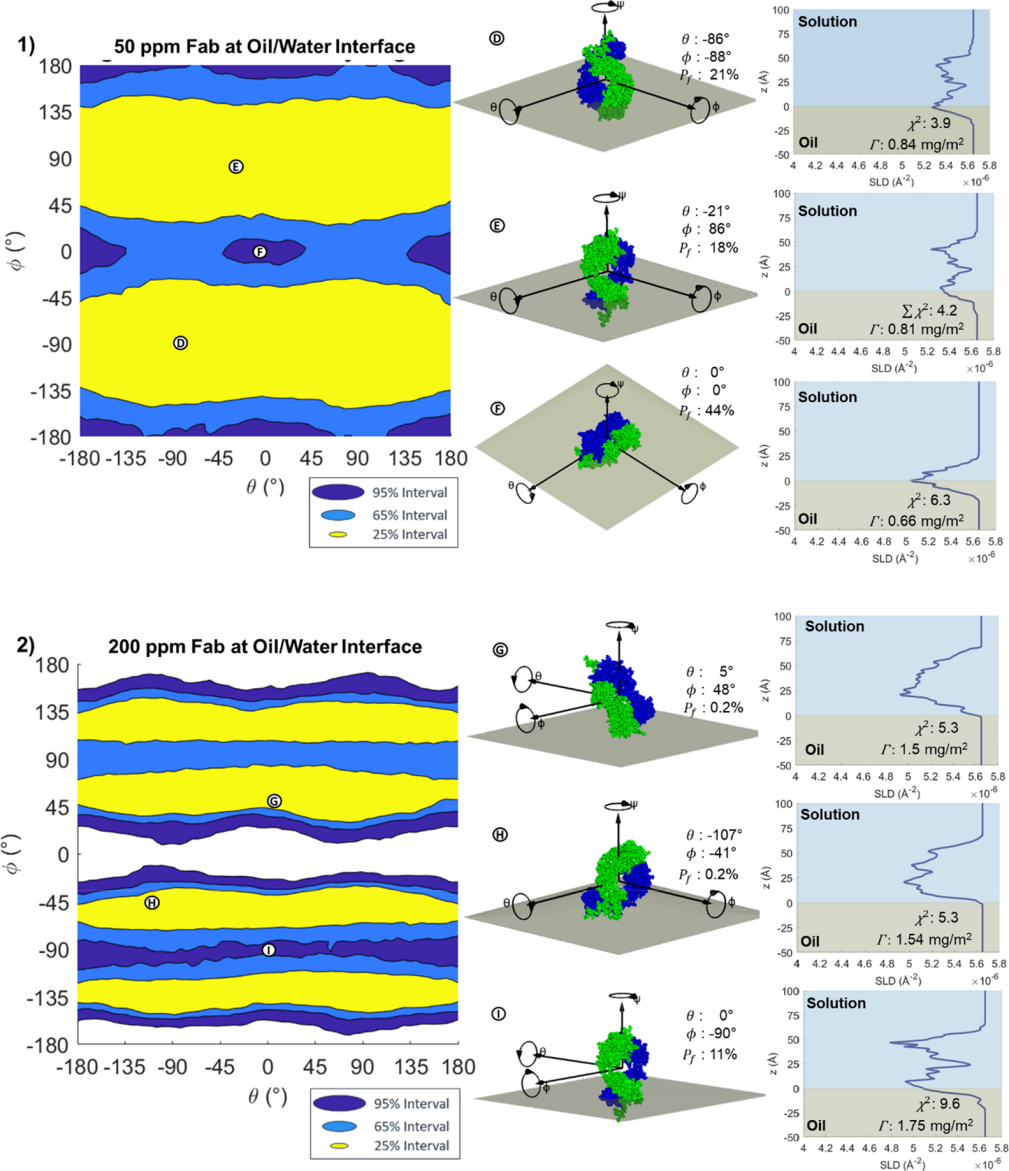
HPDI variations for the orientation of (1) 50 ppm Fab and (2) 200 ppm Fab at the oil/water interface by plotting in ϕ versus θ (left), with visualizations (middle) and SLD profiles (right) from key points labeled in the figure. SLD profiles are given in the substrate-matched aqueous contrast, in this case, CM Sapphire (SLD = 5.65 × 10^–6^ Å^–2^) to remove the contributions from the sapphire/oil and oil/water interfaces so that the entire signal arises from the adsorbed protein.

Figure 4 shows the HPDIs for the orientation of 50 and 200 ppm Fab at the oil/water interface. From the figure, it can be seen that at 50 ppm, the 95% HPDI intervals encompass the entire rotation space, demonstrating low confidence in the orientation. This low sensitivity can be seen directly in the posteriors in subfigure (a) of Figure S10, where it can be seen that the 2 million accepted Markov Chain steps fill the entire rotation space. The insensitivity of the measurement to orientation can also be observed in the change of the ∑χ^2^ value, from the best fitting of 3.9 to the worst fitting orientation of 6.3, fairly significant but not a large enough change in likelihood to limit the posterior probability to a particular region. The lower sensitivity at this interface than at the air/water interface could arise from the lower quality of NR data. Due to limitations inherent to the technique as described in the Experimental Section, the oil/water measurements were taken across a lower and narrower Q-range. An extra NR contrast was also measured for Fab in addition to NR data from previous measurements. The better fitting and higher confidence regions correspond to the fragment standing roughly on the end, with the long axis of the protein perpendicular to the interface, as seen at points Ⓓ and Ⓔ in Figure 4(1), with the regions extending to tilts roughly ±45° in ϕ from this standing on end orientation. The lower-confidence regions appear where the thickness of the protein layer is minimized, shown at point Ⓕ in Figure 4(1). This is opposite to the orientation observed at the air/water interface, but the low confidence in the orientation means further evidence is required to support the adsorbed conformation.

For the 200 ppm measurement as shown in Figure 4(2), there is far greater confidence in the orientation than at lower concentrations, consistent with the higher signal from the greatly increased amount of Fab adsorbed at the interface. The more uniformity of orientation could also arise from the denser Fab packing at the interface. At 200 ppm, there is 2.5 times more Fab at the interface than at 50 ppm, resulting in a much higher signal to noise ratio. The HPDIs for this set of measurements are also roughly uniform in θ. As with the 50 ppm measurement, the lowest probability areas, in this case outside the 95% HPDIs, appear where the Fab fragment is lying flat-on to the interface at ∼ϕ = 0°. However, the regions of higher probability are better defined, with the 25% HPDIs on either side of ϕ = + 90° and ϕ = – 90°. As shown at points Ⓖ and Ⓗ in Figure 4(2), these regions correspond to a tilted adsorbed orientation, rather than end-on or flat-on. This results in a slightly thinner SLD profile than in the end-on adsorption as seen at point Ⓘ in Figure 4(2) and without the lower volume-fraction region in the center. As these orientations are also contained within the 25% HPDIs for 50 ppm Fab, we may hypothesize that this is the preferred orientation, even though there is not sufficient sensitivity in the measurements to distinguish this. Though the structure of Fab appears roughly symmetrical along its long axis, there appears to be a difference in the probability/fitting between the two end-on orientations at ∼ϕ = + 90° and ∼ϕ = – 90°, as seen in the 65% HPDI region. This is also apparent in the KDE for the 50 ppm measurement (Figure S11c). This may be due to one end of the Fab being thicker than the other or due to its small spike on one end which could deform and not protrude if the molecule was not treated as rigid.

The outcome of this analysis is mostly consistent with our previously proposed slab model by Ruane et al., in which it was hypothesized that the Fab fragment was adsorbed in a tilted monolayer with a thickness of 59 ± 2 Å. The overall Fab envelope in the 25% HPDI region is approximately 60–70 Å thick. The rigid body model fits a higher *P*_f_ than the slab model at 50 ppm, albeit with high uncertainty, but only a slightly higher *P*_f_ in the more sensitive 200 ppm measurement.

It is apparent that the Fab takes different conformations at the air/water and oil/water interfaces despite the air/water interface traditionally being described as hydrophobic.^62^ The difference implies the lower effective hydrophobicity of the air/water interface than the oil/water interface. In addition to interfacial confinement once adsorbed, different interfacial orientations lead to different electrostatic interactions due to different local Fab surface proximities. The Fab also penetrates significantly more into the air/water interface than into the oil/water interface. For penetration into the oil phase to be energetically favorable, the region of the mAb in contact with the interface must be hydrophobic and poorly hydrated. The final orientations at the two interfaces represent the balance of all interactions together with possible local structural adjustments induced by unfavorable local influences.

### 3.2 Fc Fragment at Air/Water and Oil/Water Interfaces

**Figure 5:**
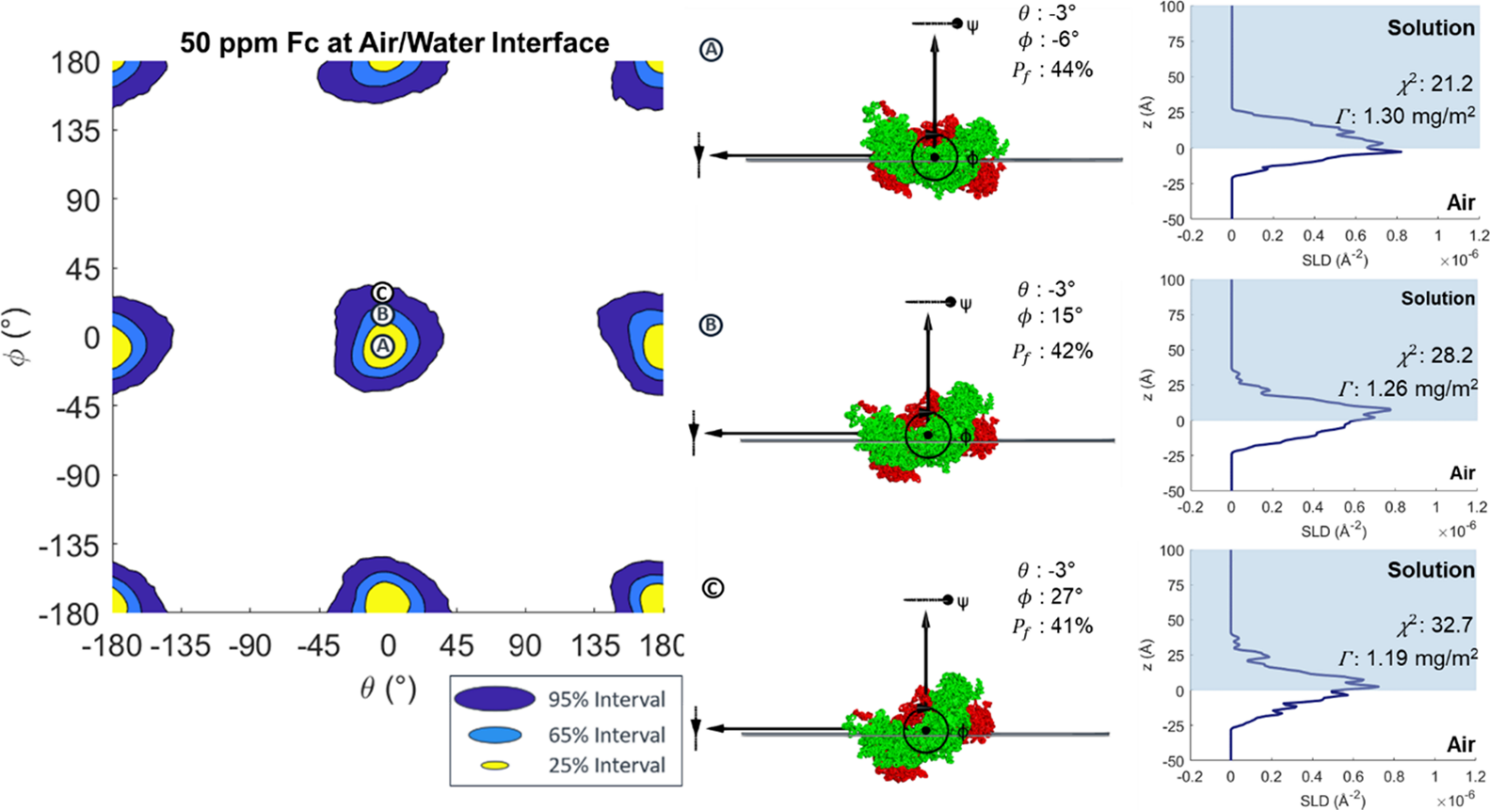
HPDI variation for 50 ppm Fc adsorbed at the air/water interface by plotting change in ϕ versus θ, (left) with visualizations (middle) and SLD profiles (right) from key points labeled in the figure.

Figure 5 shows the HPDIs for the orientation of the Fc at the air/water interface by plot change in ϕ versus θ. The intervals in Figure 5 are a set of concentric, roughly circular regions centered around θ = – 3°, ϕ = – 6°, and at points 180° away. As shown at point Ⓐ in Figure 5, the centers of these shapes correspond to the Fc fragment lying flat at the interface, minimizing the thickness of the adsorbed layer, as evident from the SLD profile. As the fragment tilts away from this point, the χ^2^ value increases, indicating a worse fit, to a maximum at ∼ ± 90° away in θ and ϕ. Points Ⓑ and Ⓒ in Figure 5 reveal how tilting the Fc broadens the SLD profile and reduces the goodness of fit. The 95% interval corresponds to a region roughly ±34° in both θ and ϕ away from the central point, and the 65% interval region is roughly ±19° and the 25% interval is around ±13°. Like Fab, the Fc prefers a flat-on, partially submerged orientation at the air/water interface. As Fc is less symmetrical around its long axis than Fab, rotating around it results in larger changes in the profile, and so there is greater sensitivity to change in θ.

The slab model analysis by Li et al. proposed a flat-on adsorption for the Fc at the air/water interface, with a total thickness of 40 Å, of which 15 Å (37.5 ± 5%) penetrated into air. Although the total protein envelope is 50–60 Å in thickness in the rigid body model, the protein is much denser toward the middle of the layer, so the models are roughly consistent, and the *P*_f_ values (34 ± 9% for the rigid body model) are very similar.

Because of the steep probability well between viable orientations, it is highly unlikely for the Markov Chain to step across into an opposite well 180° away. This makes it difficult to produce a chain that mixes well between these states, and so this chain was constructed artificially by adding together a series of separate chains started at each local minimum. Plots of the individual and combined chains can be seen in Figures S5 and S7, respectively. This issue does not occur when using fewer contrasts by, for example, taking out the CM 4.5 contrast, as the less steep probability well then allows full mixing between states. However, leaving this data out greatly broadens the confidence intervals in orientation giving a less precise result. For this reason, it is relatively easy to analyze the probability distribution of each orientation individually but comparison of the relative probability of the area centered at θ = – 3°, ϕ = – 6°, and the regions 180° away is difficult. This is a well-known problem with MCMC analysis for multimodal posteriors.^63^ Potential solutions include using more advanced MCMC methods that are less prone to this issue, such as differential evolution adaptive metropolis (DREAM)^64^ or Bayesian analysis methods that sample the probability space in a more systematic fashion, such as nested sampling.^65^ The introduction of an entirely different sampling technique for one measurement is beyond the scope of this study; however, this will be implemented in future work. Supporting figures in θ vs ϕ for the individual chain centered at θ = – 3°, ϕ = – 6° are shown in Figure S12, while supporting figures for the combined chain are shown in Figure S13.

**Figure 6:**
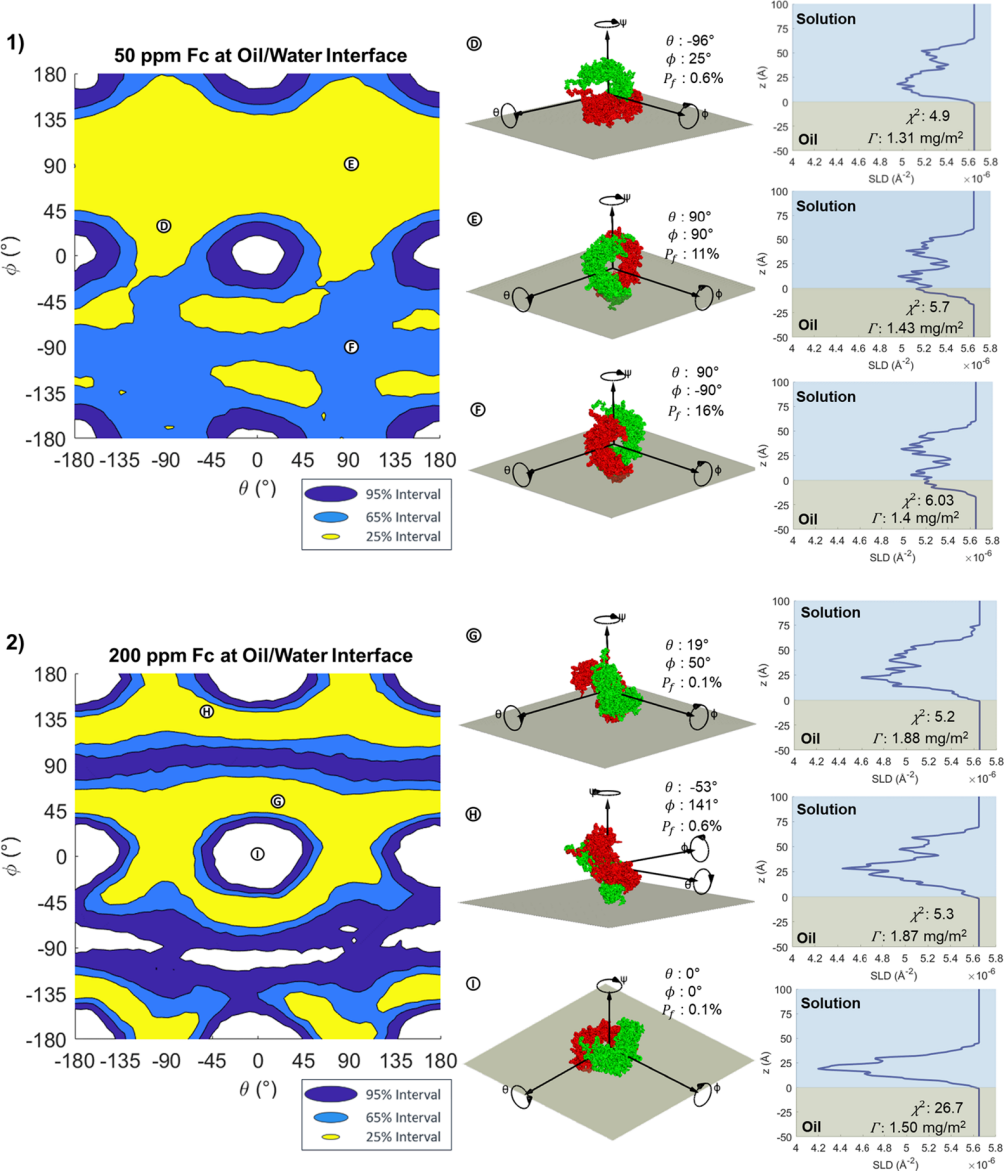
HPDI variation for the orientation of (1) 50 ppm Fc and (2) 200 ppm Fc at the oil/water interface (left), with visualizations (middle) and SLD profiles (right) of key points labeled in the figure.

Figure 6(1) shows the HDPI variation for 50 ppm Fc at the oil/water interface. Further supporting figures can be seen in Figure S14. At this interface, the 95% HPDIs exclude the areas around the points where the protein stays flat-on at the interface and minimizes the thickness of the SLD profile, with 65% intervals forming a slightly wider circle around these areas. This is roughly the opposite set of orientations to those observed at the air/water interface, where flat-on adsorption was observed. The better fitting probabilities correspond to adsorption onto the edge of the torus-like Fc fragments as shown at points Ⓓ, Ⓔ, and Ⓕ in Figure 6(1) and to adsorption where the fragment is tilted between flat-on and edge-on. As with the 50 ppm Fab adsorption, the 50 ppm Fc measurement is not highly sensitive to orientation and even the best-fitting 25% interval regions are fairly large. Interestingly, the 25% regions are not symmetrical across ϕ, as half of the figure below ϕ = 0° has slightly worse fitting orientations than the other half, as shown by comparing points Ⓔ and Ⓕ. This is likely due to the fact that the end of the Fc that includes the C-terminus of both chains is somewhat thicker than the other end, producing an asymmetry in the otherwise rotationally symmetrical protein. The data is better fit when the larger end is oriented toward the oil interface, as at point Ⓔ.

As seen with Fab, the analysis for Fc at 200 ppm (Figure 6(2) shows a much higher sensitivity to orientation than at the lower concentration. Here, the same exclusion of the flat-on and low-tilt orientations is seen, as shown at point Ⓘ in Figure 6(2). The regions outside the 95% HPDIs are wider than at 50 ppm, and the 25 and 65% intervals are much narrower. The edge-on orientations around ϕ ≈ ± 90° are also at a lower probability, with the same asymmetry as observed at 50 ppm making ϕ ≈ – 90. The best-fitting 25% intervals correspond to a tilted adsorption with a thickness of 60–70 Å, as shown at points Ⓖ and Ⓗ. Like Fab, a lower penetration is observed at 200 ppm than 50 ppm; almost no penetration is observed at 200 ppm. Again, this could arise from the increased sensitivity of the measurement to mixing between oil and water with more accurate result. Figure S15b shows that some of the region between ϕ = 0° to −180° fits to a higher *P*_f_ than ϕ = + 180° to 0° region. This may be related to why these regions have different posterior probabilities.

In our previous slab-fitting model for Fc at the oil/water interface,^19^ we fitted a two-layer model with a denser 43 Å inner layer close to the oil and a more diffuse 16 Å outer layer, hypothesizing that Fc adsorbed in a mixed fashion, with some oriented flat-on to the interface and others oriented in a tilted orientation. This model demonstrates that the data can be better explained by a monolayer of tilted Fc fragments, with two-layer reflecting changes in the density profile. Both models show a low level of interfacial penetration.

### 3.3 COE-3 at Air/Water and Oil/Water Interfaces

**Figure 7:**
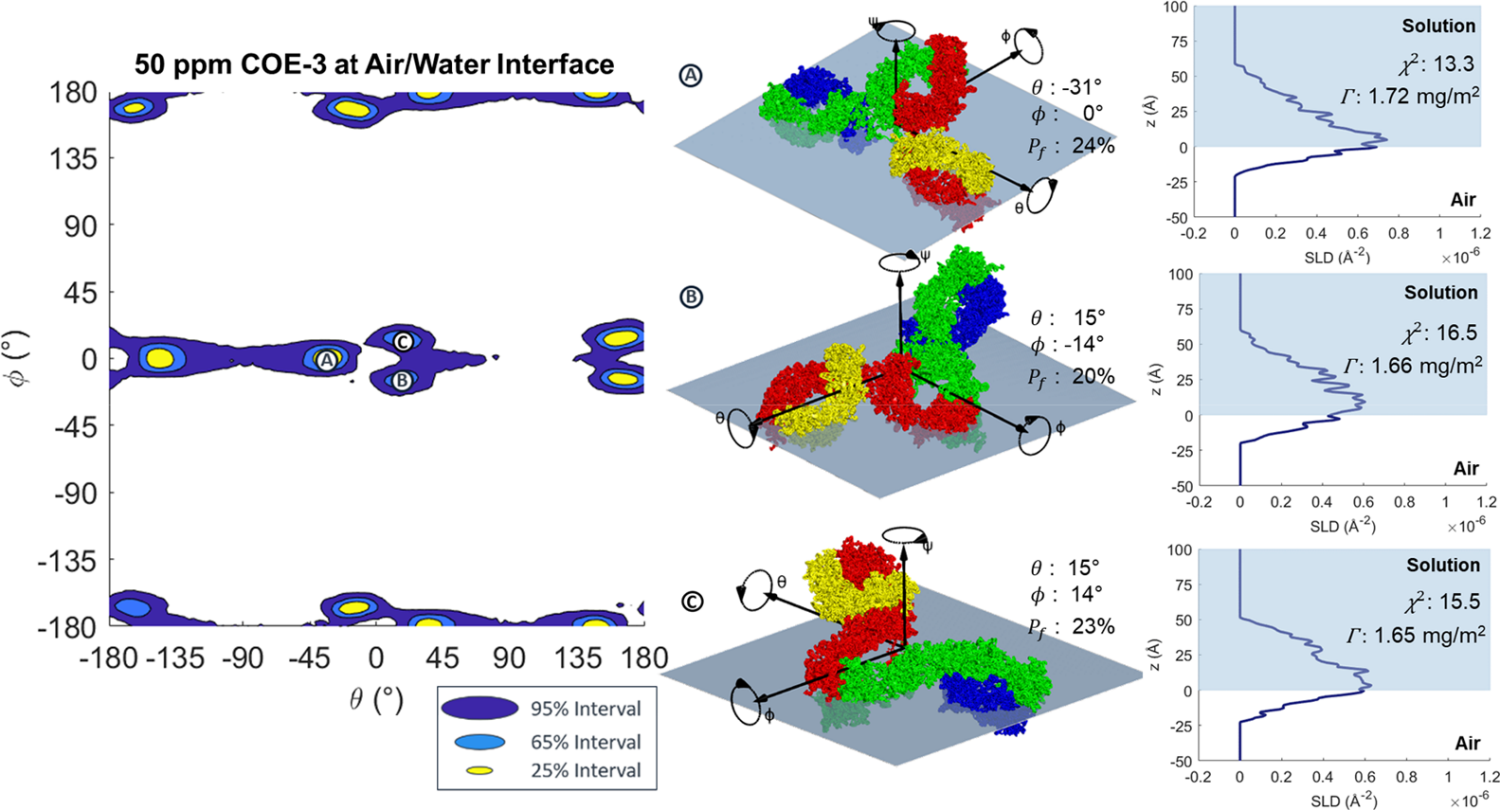
HPDI variation for the orientation of 50 ppm COE-3 adsorbed at the air/water interface (left), with visualizations (middle) and SLD profiles (right) from key points labeled in the figure.

Figure 7 shows the HPDI variation to depict the orientation of COE-3 at the air/water interface, along with representative orientations. Further supporting figures can be seen in Figure S16. Comparing the HPDI plots for Fab and Fc, the posterior intervals occupy a relatively small area of the probability space, demonstrating a high sensitivity to the orientation of the protein. Due to the highly non-spherical geometry of COE-3, a small change in orientation will greatly affect the SLD profile, to a greater extent than spherical proteins such as Fab and Fc. The large uncertainty in the SLD profiles for COE-3 in Figure S1 demonstrates that even these small regions in orientation correspond to large ranges in the SLD profile.

Across the probability profile, the highest probability regions adopt a repeated ‘*Y*’ shape, most clearly near (θ = 0°, ϕ = 0°). This shape is similar to the overall profile of the protein, and indeed, each lobe of the ‘*Y*’ corresponds to a tilted orientation with one of the fragments protruding away from the interface. Examples can be seen at point Ⓐ in Figure 7, which corresponds to the Fc fragment tilting away, and points Ⓑ and Ⓒ, which correspond to one of the Fabs being tilted. Although it is possible to distinguish these states, similarities in the resulting SLD profiles make it difficult to identify them. We can however hypothesize that as the Fc has a greater affinity to the surface than Fab (due to the higher adsorbed mass at each concentration), it is less likely to be tilted away. It is also likely that the actual layer is a combination of both, with some mAbs oriented with Fabs away from the interface and some oriented with Fcs, resulting in an average SLD profile with evident mAb tilting. Some orientations with two fragments protruding from the interface, corresponding to the gaps between the peaks of the ‘*Y*’, are within the 95% confidence interval, but these have a lower probability density than the orientations with one protruding fragment.

**Figure 8:**
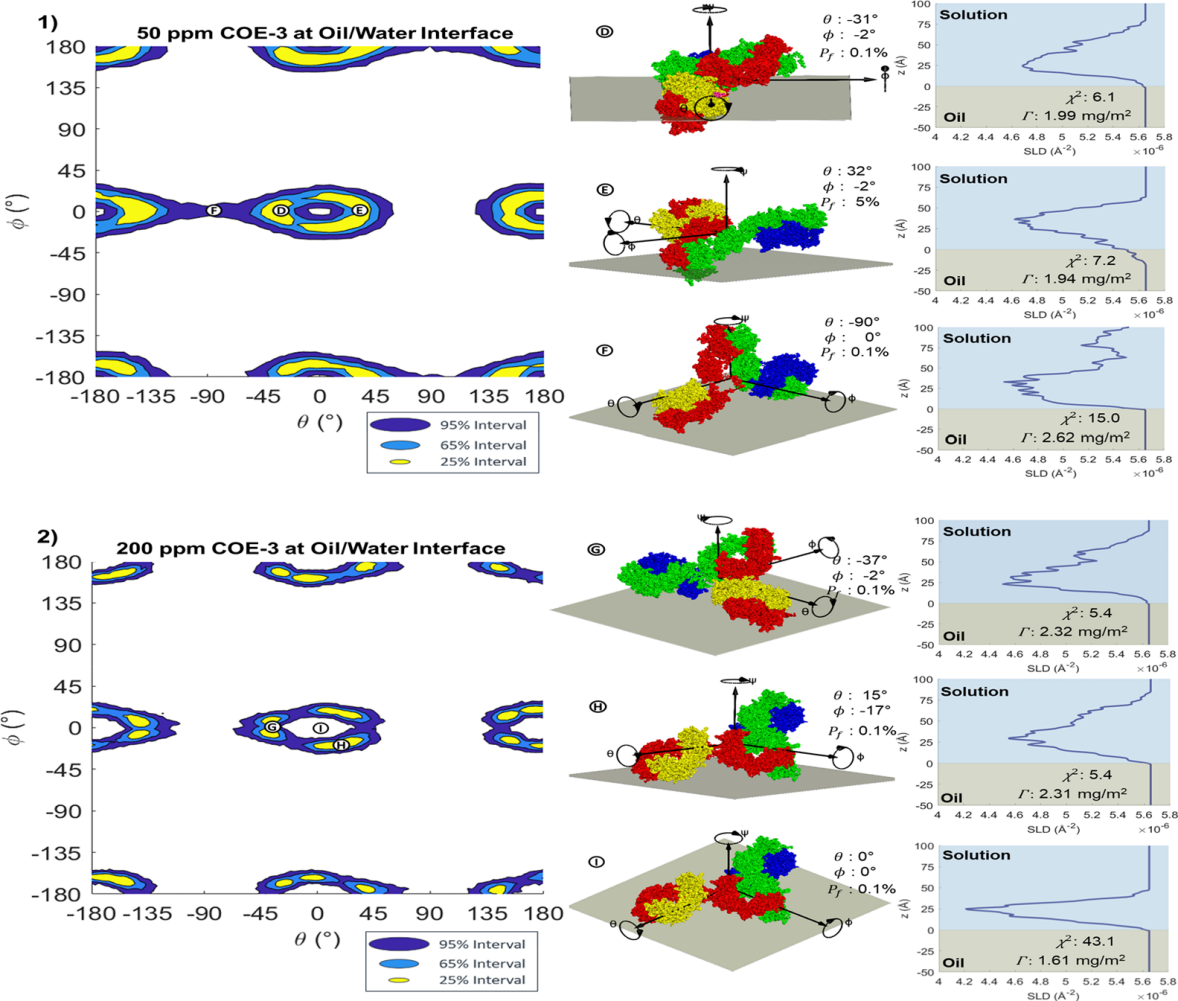
HPDI variation for the orientation of (1) 50 ppm Fab and (2) 200 ppm COE-3 at the oil/water interface (left), with visualizations (middle) and SLD profiles (right) of key points labeled in the figure.

The HPDI variation plots for COE-3 at the oil/water interface are displayed in Figure 8. Overall, these figures show similar credible intervals to COE-3 adsorption, forming a region around but excluding the center. This again corresponds to a tilted orientation at the interface, with one or two fragments protruding from the interface. As with Fc and Fab, the data at 50 ppm has much less specific confidence intervals than the 200 ppm case, with even the 25% HPDI forming an oval rather than a ‘*Y*’. This indicates insensitivity to the direction in which the mAb is tilted, with either one fragment or two fragments tilting away from the oil, as in points Ⓓ and Ⓔ (in Figure 8(1)) respectively. Point Ⓕ in the same figure shows that the 95% confidence interval also includes the “head-on” orientation, with both Fabs contacting the oil and the mAb tilted perpendicular to the interface, a significantly different SLD profile from the tilted adsorption. The 200 ppm measurement shows a greater sensitivity, demonstrating intervals similar to the *Y* shape displayed at the air/water interface, where the 25 and 65% confidence intervals again occur with one fragment protruding from the interface, as shown at points Ⓖ and Ⓗ in Figure 8. The intervals at the oil/water interface demonstrate a larger tilt than at the air water interface, suggesting that protein is protruding further into the solution. The much higher sensitivity to orientation at 200 ppm despite a relatively small increase in the adsorbed mass (2.06 ± 0.20 mg/m^2^ at 50 ppm to 2.40 ± 0.20 mg/m^2^ at 200 ppm) suggests an increased homogeneity of the orientation of the protein upon denser packing at higher concentrations. Further supporting figures for the 50 and 200 ppm measurements can be seen in Figures S17 and S18 respectively.

However, the protein rotation model does not allow for flexibility of mAb, especially at the disulfide hinge regions, while proteins and their globular fragments are often flexible in aqueous solution.^66^ This greatly restricts the possible conformations the mAb could take at the interface and does not facilitate the exploration of all possible SLD profiles. For example, rotation of the fragments relative to the overall mAb could be important, with some of the fragments adopting the same orientation as when adsorbed. Similarly, a tilted orientation can be different to the previous slab-based analysis at the air/water interface as proposed by Li et al. assuming that the mAb molecule lay flat against the interface, with Fabs protruding further out from the interface than the Fcs due to a more side-on orientation.^9^ From the rotation analysis, this seems less likely, as both fragments appear to prefer a flat-on orientation at the interface. However, it is possible that in the full mAb, the Fc is constrained from adopting its preferred orientation. This aspect could be explored by using a NR data analysis program that allows flexibility in the molecule, such as SASSIE’s SLDmol.^67,68^ The rigid body analysis at the oil/water interface is consistent with the previous slab analysis, in which it was hypothesized that some mAb fragments were adsorbed in a tilted manner, forming a more diffuse outer layer.

Despite the potential for variability in the structure used due to the flexibility of COE-3, the χ^2^ values for the best fitting orientations shown in Table S2 are lower than those from the slab fit model despite being constrained by the structure. This suggests that even if the average structure adopted by COE-3 is significantly different to that input into the rotation model, the overall concept of the fit, with one fragment protruding into/away from the interface, is representative of the mAb layer. The results also suggest that at the very least the SLD profiles generated by the protein rotation will be a useful representation of protein’s density profiles. As we have tied the profiles directly to the structures, we can have greater confidence than possible with the slab model because the adsorbed layer can be directly described as a tilted monolayer instead of abstract layers that could be tilted proteins or multiple adsorbed monolayers.

### 3.4 Changes in Area per Molecule (APM)

Table S1 gives the average footprint APM values and errors for each protein under different conditions calculated from the rigid body model using both concave hull (CH) and convex boundary (CB) methods, and also calculated from Γ. Figure S1 shows examples of how these values are calculated from the orientation. While Fab and Fc have relatively similar values from the CH and CB methods, the values are substantially different for COE-3. The CH method is more commonly used in MD analysis for globular proteins to describe molecular footprints. Unlike Fab and Fc, COE-3 is substantially concave in shape, so the CH method will produce a large overestimate for the APM when the molecule is flat-on. Figures S2–S4 show how the calculated area per molecule changes with orientation for Fab, Fc, and COE-3, respectively. Comparing with the HPDI plots for their respective proteins, it can be seen that the intervals tend to follow areas of similar APM, especially for Fab and Fc.

Because of their flat-on orientation, CH and CB APM values for the Fab and Fc fragments at the air/water interface are close to their maximum possible values (CH = 3100 Å^2^ , CB = 2800 Å^2^ for Fab; CH = 3500 Å^2^ , and CB = 3300 Å^2^ for Fc). The values for the APM calculated from Γ are significantly higher at 200 ppm. This suggests that there is space for every protein at the interface and that tilted/end-on adsorption of Fab and Fc at the oil/water interface is not caused by direct physical blocking/steric overlap of protein adsorption sites, especially as the tilted orientation is still observed in the low surface coverage measured at 50 ppm. This provides further evidence that the tilted adsorption is driven by surface interactions with regions of the protein, that is, hydrophobic or charged interactions with the oil. Comparing the CB-projected APM to the measured APM, COE-3 covers the highest proportion of the available space, 61% at the air/water interface and 75% at 50 ppm at the oil/water interface. This may explain why COE-3 adsorbs in a tilted orientation at both interfaces, despite both its component fragments individually having an affinity for the interface. As a flexible molecule, some of the fragments may adsorb to the interface while others may even lie on top of the monolayer.

## 4 Conclusions

This study has demonstrated that the rigid body modeling of NR data can provide a robust analysis of some globular proteins adsorbed at interfaces, while applicability to hinged proteins remains to be fully demonstrated. Fc and Fab were both found to adopt significantly different orientations at the oil/water interface. In contrast, they are adsorbed in a flat-on orientation to the air/water interface with minor tilting. The difference may well arise from different substrate/protein interactions which are of electrostatic and hydrophobic origins, instead of steric blocking of adsorption sites at the interface. These differences are made very explicit by the rigid-body model, while in the slab model, they can only be loosely inferred from changes in layer thickness. All measured neutron reflectivity data were well-fit with a monolayer of adsorbed protein, suggesting no major deformation or unfolding of the protein. The adsorbed Fc layer was better explained by a monolayer of tilted molecules, giving greater structural insights into the previously hypothesized two-layer model. In contrast, COE-3 was found to adopt a similar tilted orientation at both interfaces, with two fragments in contact with the interface and one protruding into the solution. This study shows that the rigid body analysis can be a useful tool to analyze NR data for the adsorption of globular proteins. Future work could focus on an assessment of flexibility of the protein,^67^ in order to explore potential conformations of the mAb.

High quality NR data, with large *Q*-range, low measurement error, and multiple aqueous contrasts were found to be important in allowing sensitive analysis of orientation. Increased concentration of protein was also found to increase sensitivity, by either increasing the signal-to-noise ratio or by increasing layer homogeneity. Comparing the area per molecule calculated from the orientation with that calculated from Γ offers a useful route in assessing structural flexibility and packing of the protein molecules in the adsorbed layer. The difference in sensitivity to orientation between Fab and Fc also demonstrates that less radially homogeneous proteins are more suitable for rigid body analysis. A greater sensitivity to orientation could potentially be achieved in future studies from deuterated proteins, as this would provide another contrast and sensitivity to protein orientation. Further sensitivity could be gained by using partially deuterated mAbs, for example, a single mAb region or chain deuterated. This would break the rotational symmetry of the protein, providing specific sensitivity that would allow, for example, discrimination between 180° rotated states for Fab and Fc and discrimination of which the fragment was likely protruding into/away from the interface for COE-3.

Given the advantageous features over slab modeling, this study has demonstrated the power of rigid body modeling for structural analysis at other interfaces or of more complex adsorption experiments, such as exploring how polysorbate surfactants affect orientation and adsorption behavior of mAbs.

## Supporting Information Available

Neutron reflectivity profiles and best model fits, key parameters from data analysis, and area per molecule calculations and orientations of Fab, Fc, and the whole COE-3 at the air/water and oil/water interfaces (PDF)
