# Supplementary material for: Investigating the Orientation of an Interfacially Adsorbed Monoclonal Antibody and Its Fragments Using Neutron Reflection
Source: Mol Pharm. 2023 Feb 16;20(3):1643–56. doi: 10.1021/acs.molpharmaceut.2c00864 (PMC9996827; doi:10.1021/acs.molpharmaceut.2c00864)
Supplement: Supplementary file 1 — mp2c00864_si_001.pdf [file mp2c00864_si_001.pdf]

Supporting Information

Investigating the Orientation of an Interfacially Adsorbed Monoclonal Antibody and Its Fragments Using Neutron Reflection

Sean Ruane[1], Zongyi Li[1], Peter Hollowell[1], Arwel Hughes[2], Jim Warwicker[3], John R. P. Webster[2], Christopher F. van der Walle[4], Cavan Kalonia[5], Jian R. Lu[1*]

[1] Biological Physics Laboratory, School of Physics and Astronomy, University of Manchester, Oxford Road, Schuster Building, Manchester M13 9PL, UK.

[2] ISIS Neutron Facility, STFC, Chilton, Didcot OX11 0QZ, UK.

[3] Division of Molecular & Cellular Function, Manchester Institute of Biotechnology, University of Manchester, Oxford Road, Manchester M13 9PL, UK.

[4] Dosage Form Design & Development, Biopharmaceutical Development, AstraZeneca, Cambridge, CB21 6GH, UK.

[5] Dosage Form Design & Development, AstraZeneca, Gaithersburg, MD 20878, USA.

*Corresponding author: Jian R Lu (email: j.lu@manchester.ac.uk; Tel: +44 161 2003926)

Section 1 Area Per Molecule Calculations

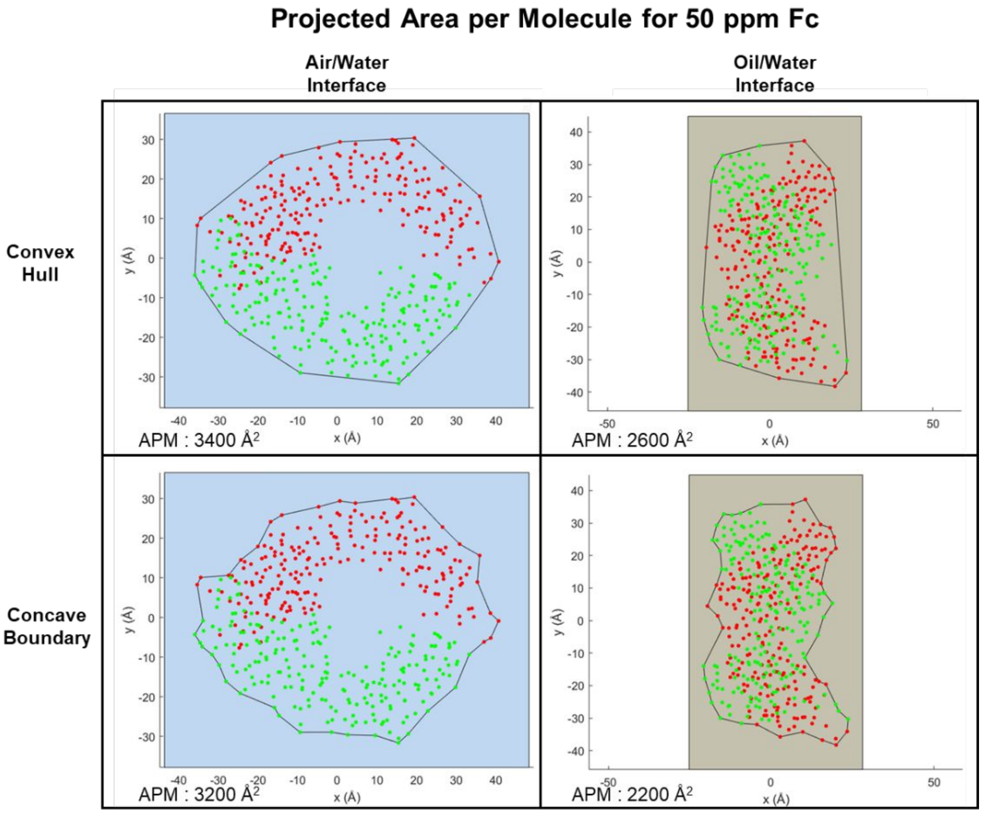

Figure S1. Example of how the convex hull and concave boundary footprints are calculated using the best fitting conformations of 50 ppm Fc at the air/water and oil/water interfaces (see Figures 5 and 6.1 in main text). The perspective is taken from above the protein, with the z axis into the page.

Table S1 gives the average area per molecule calcualations for each protein in each condition.

Protein	Interface	Concentration (ppm)	Area Per Molecule (Å^2) Convex Hull	Concave Boundary	Calculated from Γ
Fab	Air/Water	50	3000 ± 100	2600 ± 100	10000 ± 1400
	Oil/Water	50	2200 ± 500	2000 ± 400	12100 ± 3900
		200	2200 ± 300	2000 ± 300	4900 ± 500
Fc	Air/Water	50	3400 ± 100	3200 ± 100	6800 ± 500
	Oil/Water	50	2600 ± 300	2200 ± 300	6400 ± 600
		200	2700 ± 300	2300 ± 300	4300 ± 300
COE3	Air/Water	50	12300 ± 300	8800 ± 300	14300 ± 1000
	Oil/Water	50	12500 ± 300	8800 ± 300	11700 ± 1100
		200	12300 ± 300	8800 ± 300	10100 ± 800

Table S1. Average APM calculations for each protein from Convex Hull, Concave Boundary and measured directly from the adsorbed mass.

Demonstrations of how convex hull and concave boundary values were calculated are shown in Figure S1 above, using the Fc fragment as an example. The measured APM from Γ was calculated using equation 9 in the main text. To reduce the necessary computation time, convex hull and concave boundary calculations were performed for every 10th chain step. Convex hull values were calculated using the quickhull algorithm [4]. Concave boundary values were calculated using MATLAB's boundary function, an implementation of an Alpha shapes boundary function, with a shrink factor of 0.5 [5,6]. Errors for concave hull and convex boundary were calculated by taking the standard deviation of all values calculated, while errors for APM calculated from Γ were propagated from errors in adsorbed mass.

Figure S2 shows how the APM calculated via the a) convex hull and b) concave boundary of changes with the orientation of the Fab fragment. Comparing these plots to the HPDI plots in the main text and the probability plots below demonstrates a strong correlation between projected APM and goodness of fit. The convex hull calculation appears to result in a smoother plot. Figures S3 shows the same calculations for the Fc fragment, and Figure S4 shows the same for the full-length COE-3.

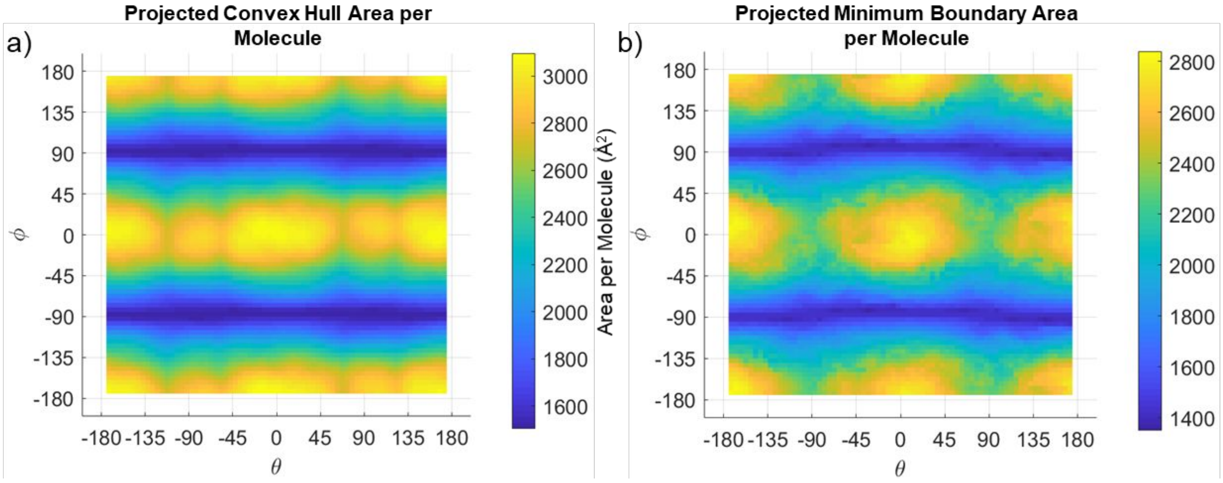

Figure S2. APM values for the Fab fragment calculated by a) convex hull and b) concave boundary

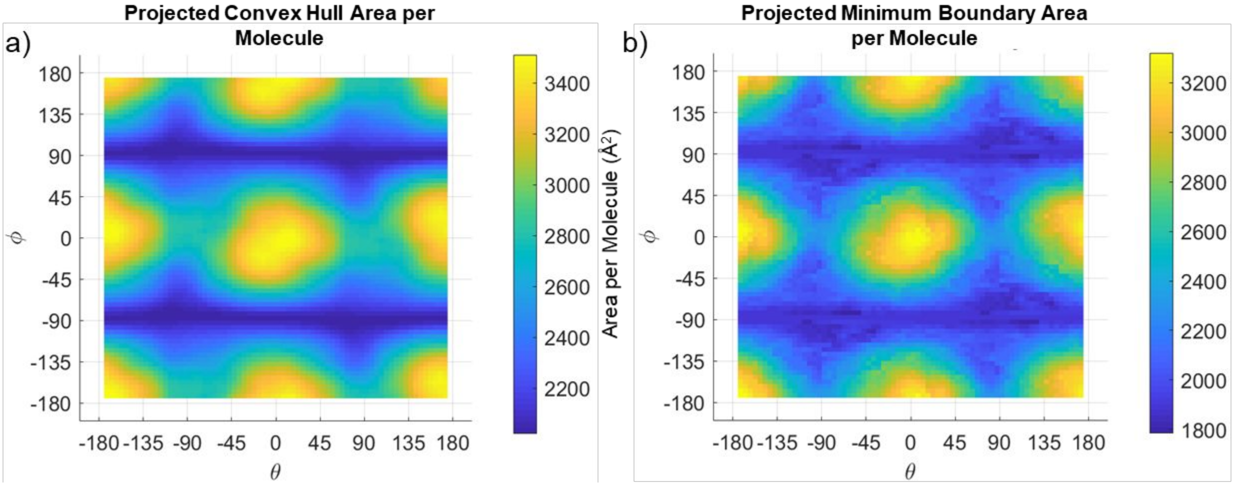

Figure S3. Area per molecule (APM) values for the Fc fragment calculated by a) convex hull and b) concave boundary methods.

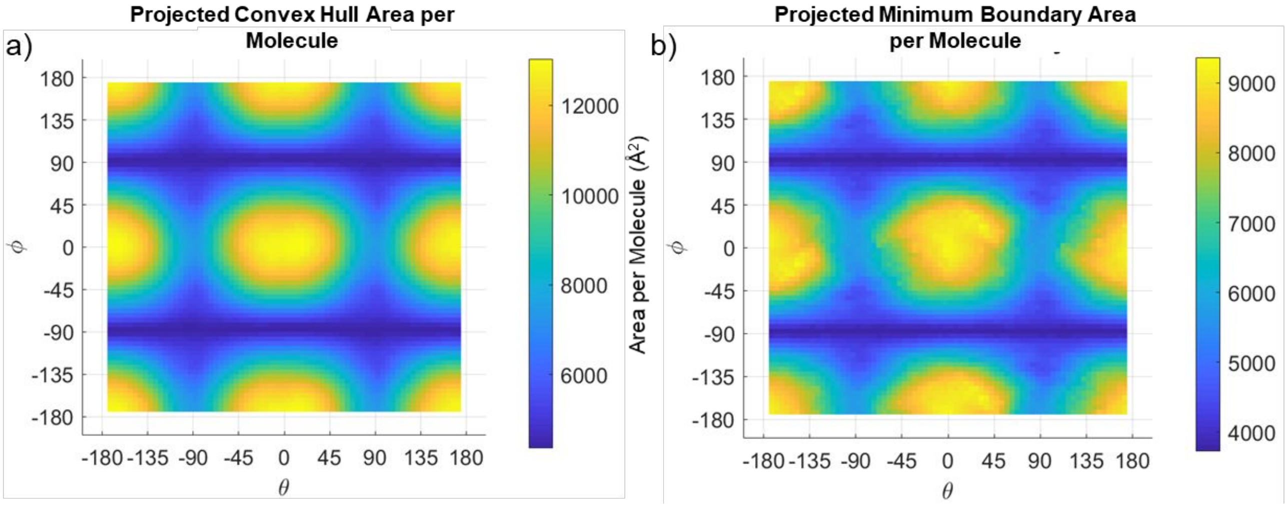

Figure S4 APM values for COE-3 calculated by a) convex hull and b) concave boundary methods.

Section 1 MCMC Diagnostics

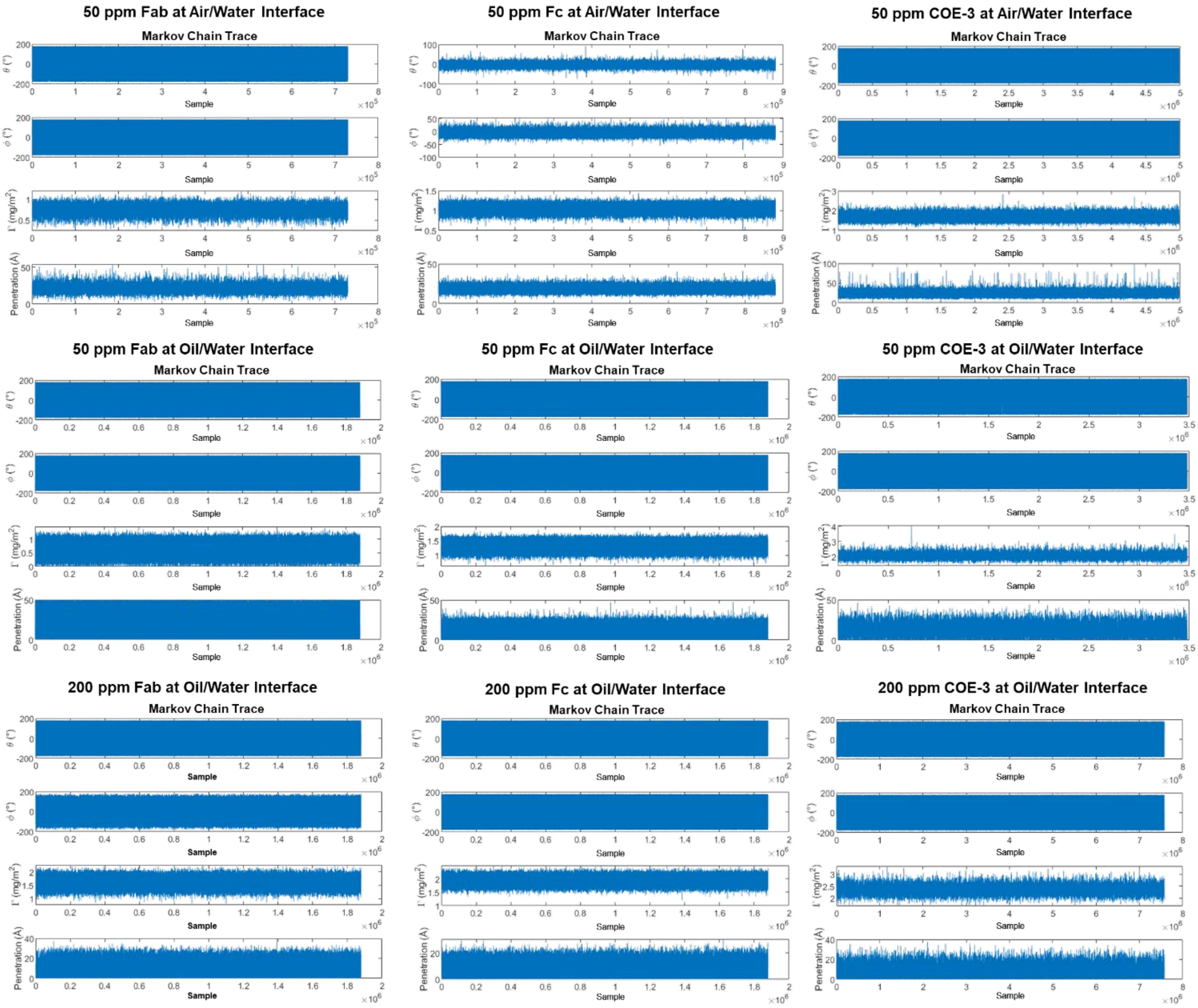

Figure S5. Trace plots for each MCMC chain. These plot show how the values of each fitting parameter evolve throughout a MCMC run by plotting the parameter value for each accepted step sequentially. For the Markov Chain to sample the posterior probability effectively, the average value should not change significantly throughout the course of the run. The above traces show good mixing, with the chain stepping quickly across the whole parameter space, rather than "sticking" in particular regions.

Figure S5 shows the behaviour of the MCMC for each variable over time. It is important to be aware of the fact that MCMC chains can often behave in biased fashion in their initial states, before the variables begin to iterate fully, and thus general a set of initial "burn-in" points are removed from the dataset. Though there is debate about whether the requirement of "burn-in points" simply suggests an insufficiently long MCMC run [7], with limited computational resources discarding runs pre-divergence can be important to ensure that the initial state is not privileged. The plots shown are with the burn in points removed.

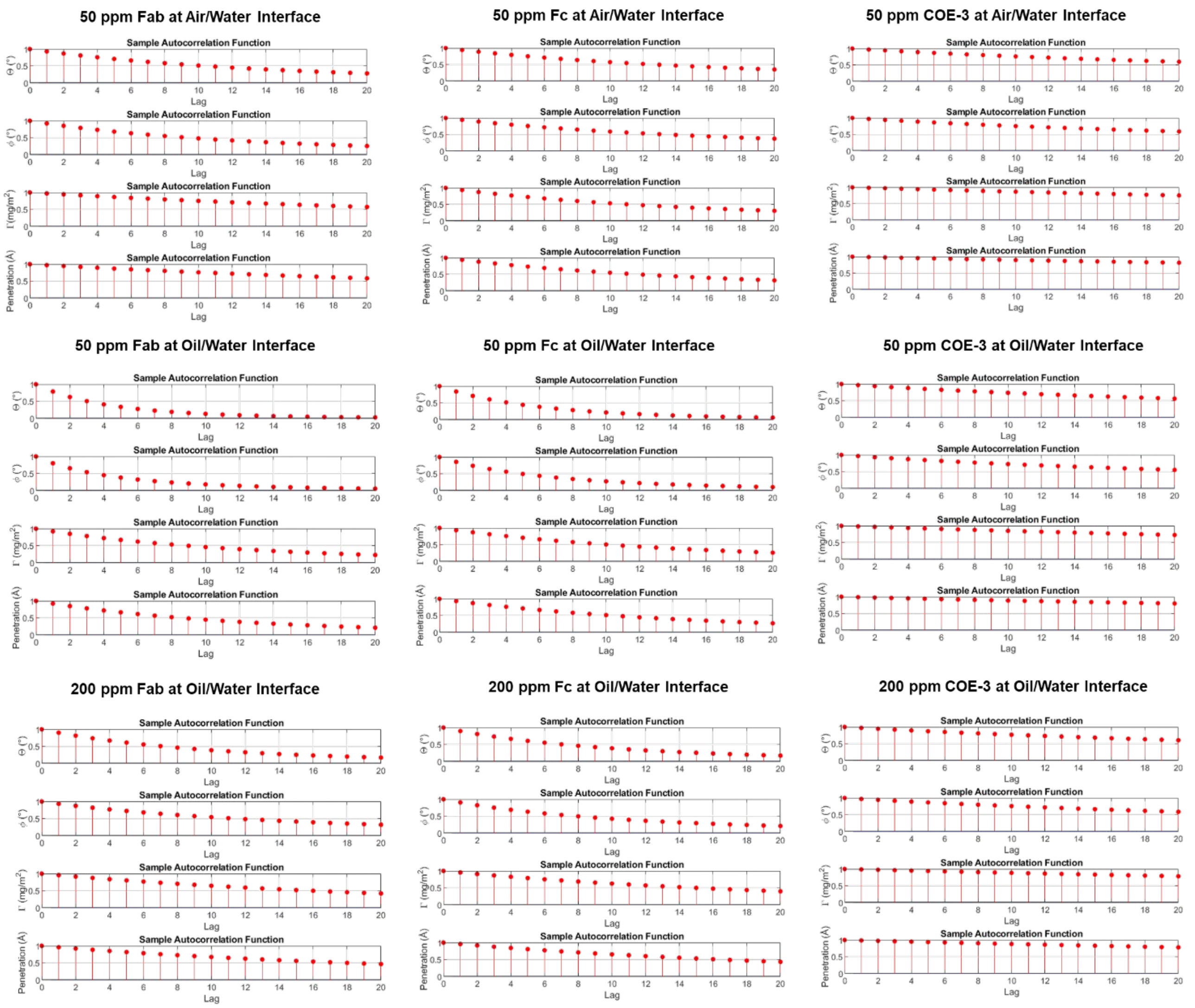

Figure S6. Autocorrelation plots for each fitting parameter for each MCMC chain. Plots the average correlation between a step and the step (lag) steps prior, giving an indication of how dependent a step is on the steps prior to it. Ideally, for proper sampling of the posterior distribution, the step should not be dependent on the steps prior to it and so the corelation should decay quickly. Correlation for COE-3 results were calculated after thinning of chains.

A large proportion of the steps for each of the COE-3 MCMC runs were rejected, requiring longer runs to produce chains of adequate length. From figure S6 it can be seen that the autocorrelation for the COE-3 posterior distributions is significantly higher than those for Fab and Fc, meaning the value of a step is more dependent on the preceding steps than would be ideal. This autocorrelation was reduced by “thinning” of the chains, taking only every n^{th} accepted step, and doing so results in similar posteriors with a lower autocorrelation. Thinning is considered slightly less accurate than using a longer chain, even if autocorrelated, but was used here due to time constraints when processing longer runs [8]. Initial chains for

COE-3 were approximately 8 times longer than for Fab and Fc runs, and were thinned by factor 8, resulting in similar chain lengths.

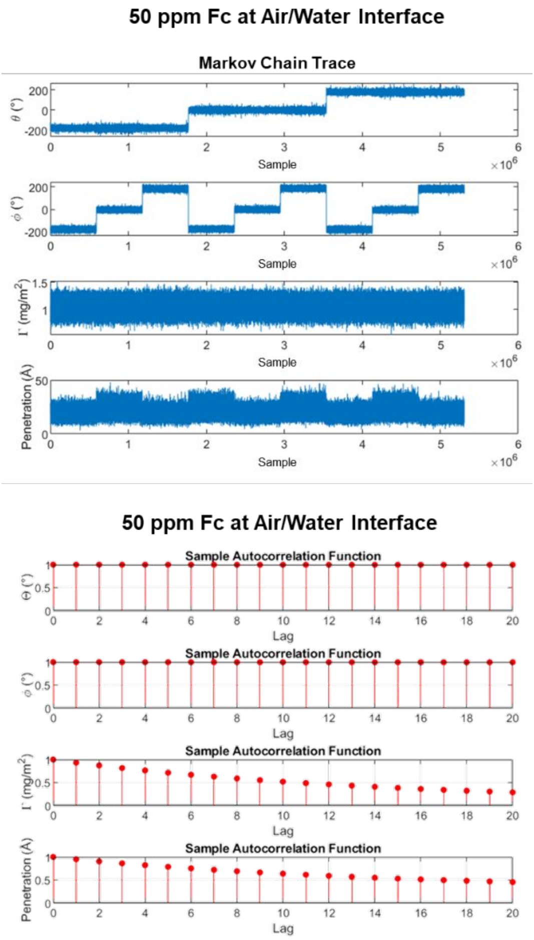

Figure S7. Trace and autocorrelation for the composite chain for 50 ppm Fc at the air/water interface.

Figure S7 shows the diagnostic plots for the composite chain for 50 ppm Fc at the Air/Water interface. As described in the main text, this chain was “stitched” together from several individual chains. Each component chain was ran for 1 million steps, starting from one of the local minima. From the autocorrelation plot, it can be seen that the θ and ϕ parameters are highly autocorrelated, due to the chain remaining in the local minima in which it was started.

Section 1 Neutron Reflectivity Data and SLD Profiles

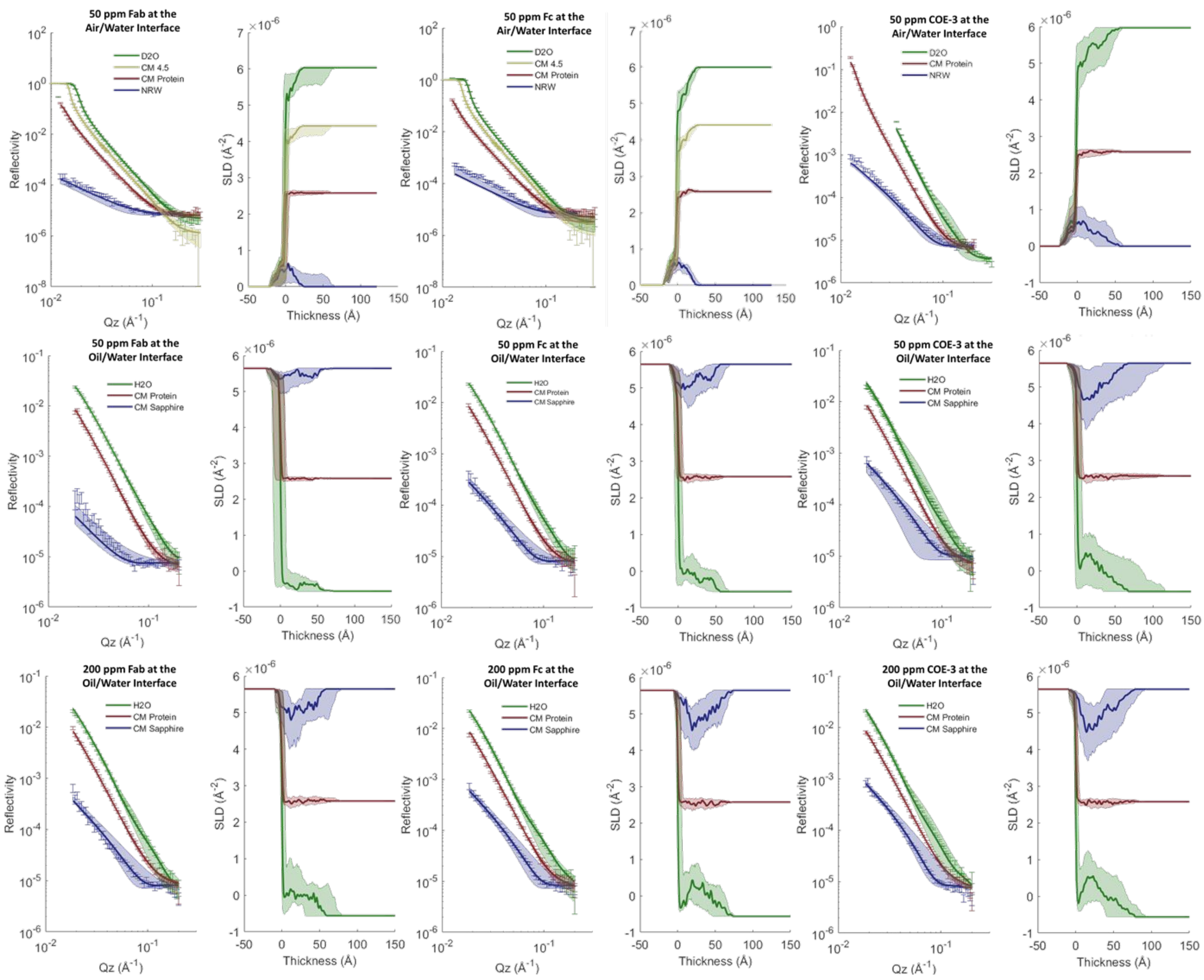

Figure S8. Neutron reflectivity profiles with the best fits for the Rigid Rotating Body Model shown as continuous lines for Fab, Fc and the whole mAb COE-3 adsorbed at the air/water and oil-water interfaces, with the exact protein concentration and isotopic contrast conditions indicated in each figure. The corresponding SLD profiles from the best first are also shown, with the shaded areas corresponding to the 65% confidence intervals. The oil used was hexadecane and all aqueous solutions were at pH 5.5 using HIS buffer, with all NR measurements being undertaken at 20-22 ^{o}C.

The experimentally measured neutron reflectivity profiles under 3 different isotopic contrasts together with the best fits are shown in Figure S1 covering the adsorption of Fab, Fc and the whole mAb COE-3 at both air-water and oil (hexadecane)/water interfaces under different protein concentrations. The best-fitted SLD profiles for each of the proteins at each interface and concentration are also shown as continuous lines, along with their 65% confidence intervals. For each condition, confidence intervals were determined by taking a sample of 3000 chain steps and calculating the confidence intervals on their respective parameters resulting in a set of SLD profiles and simulated reflectivity data. A 2D interpolation of the set

of results was created and used to find the 65th percentile result for each Q_z value for the reflectivity data and each z value for the simulated SLD profile. This capability is built in to RasCAL [1].

Section 2 Best fitting Parameters

Air/Water		Rigid-Body Rotation Model					Slab Model					
							Layer in Water		Layer in Air			
Protein	Concentration (ppm)	θ	φ	Penetration (Å)	Γ (mg/m^2)	Sum χ^2	Thickness (Å)	Protein Fraction	Thickness (Å)	Protein Fraction	Γ (mg/m^2)	Sum χ^2
Fab	50	1.9	-2.4	19	0.78 ± 0.11	9.6	40	0.16	10	0.16	1.16	35/11*
Fc	50	-3.5	-8.1	19	1.21 ± 0.09	21	25	0.29	15	0.29	1.61	120/36*
COE-3	50	-32	0	21	1.69 ± 0.13	13.3	40	0.29	10	0.3	2.01	33

Oil/Water		Rigid-Body Rotation Model					Slab Model					
							Inner Layer		Outer Layer			
Protein	Concentration (ppm)	θ	φ	Penetration (Å)	Γ (mg/m^2)	Sum χ^2	Thickness (Å)	Protein Fraction	Thickness (Å)	Protein Fraction	Γ (mg/m^2)	Sum χ^2
Fab	50	144	95	18	0.65 ± 0.21	3.9	59	0.1			0.78	5.8
	200	179.8	131	0.1	1.6 ± 0.17	5.3	59	0.2			1.58	8.4
Fc	50	-94	27	2.1	1.3 ± 0.13	4.9	43	0.19	21	0.09	1.29	5.9
	200	163	127	1.9	1.92 ± 0.14	5.1	44	0.27	16	0.18	2.01	7.3
COE-3	50	34	-177	0.8	2.07 ± 0.2	6.1	46	0.29	17	0.12	2.06	8.8
	200	-19	-165	0.1	2.4 ± 0.2	6	45	0.3	20	0.12	2.4	11.1

Table S2. Parameters obtained from the best-fitting rigid-body rotation models and slab models, with the resulting χ^2 goodness-of-fit. Parameters obtained for air/water slab models were taken from Li. et al. [2]. Parameters for oil/water slab models taken from Ruane et al. [3].

Table S2 shows the best-fitting parameters for the rigid-body rotation model for each protein at each interface and concentration. These are compared to the best parameters as previously published in works using slab modelling. Average values and errors are not given for θ and φ as the monovariate error is not particularly meaningful, due to the rotational symmetry and multimodal distribution of the posteriors. For example, examining Figure 6.1) in the main text for 50ppm Fc at the Oil/Water interface, we would expect a mean θ value of ~ 0° $\pm$ 90° as the problem is roughly symmetrical. Errors in Γ were determined by taking the standard deviation for all values in the chain.

Section 3 Penetration into Interface

Air/Water		Rigid-Body Rotation Model		Slab Model	
Protein	Concentration (ppm)	Penetration (Å)	P_f	Penetration (Å)	P_f
Fab	50	21.2 ± 5.3	0.4 ± 0.14	10 ± 2	0.25 ± 0.03
Fc	50	18.8 ± 3.6	0.34 ± 0.09	15 ± 2	0.375 ± 0.05
COE-3	50	23.2 ± 7.4	0.21 ± 0.07	10 ± 2	0.25 ± 0.03

Oil/Water		Rigid-Body Rotation Model		Slab Model	
Protein	Concentration (ppm)	Penetration (Å)	P_f	Penetration (Å)	P_f
Fab	50	18.7 ± 11.2	0.21 ± 0.19	3.5 ± 4.5	0.06 ± 0.08
	200	8.5 ± 5.8	0.05 ± 0.05	0.5 ± 2.5	0.01 ± 0.05
Fc	50	11.4 ± 6.3	0.1 ± 0.08	2 ± 3	0.04 ± 0.06
	200	4.9 ± 2.8	0.03 ± 0.02	0.5 ± 1.5	0.01 ± 0.03
COE-3	50	13.2 ± 7.7	0.06 ± 0.05	0.5 ± 1.5	0.01 ± 0.03
	200	9.6 ± 6	0.03 ± 0.03	0.5 ± 0.5	0.01 ± 0.01

Table S3. Average penetration and P_f values for each protein at each concentration and interface, with the values for the rigid-body rotation models compared to the previously published slab models.

Table S3 shows the average values and errors for the interfacial penetration for each protein into each respective interface and the resulting average P_f values, the fraction of the protein penetrated into the substrate. Errors in penetration were determined by taking the standard deviation of all the chain values, errors in P_f were propagated from these values.

Section 5 Supporting Orientation Figures

The following sections show supporting figures that are plotted in orientation space, θ vs ϕ, for each protein. These are plotted together for ease of comparison. For each interface/concentration, subplot a) shows the raw posterior points in θ vs ϕ, the θ and ϕ value for each accepted step. Plot d) is a probability histogram, found by dividing the space into 5° by 5° bins, then dividing the number of points in each bin by the total number of points to give a probability density. Plot c) is a kernel density estimate, where a Gaussian kernel is propagated from each point and the resulting probability across the space calculated. The bandwidths used for the kernel, are given in the figures, and the height of the kernel is scaled such that the sum of the resulting probability is one. This technique retains information about the location of the individual points, which is lost when binning into a histogram. Plot d) is the average P_f value for each 5° by 5° histogram bin, demonstrating how the penetration behaviour changes with rotation.

Section 5.1 Fab Fragment

50 ppm Fab at Air/Water Interface

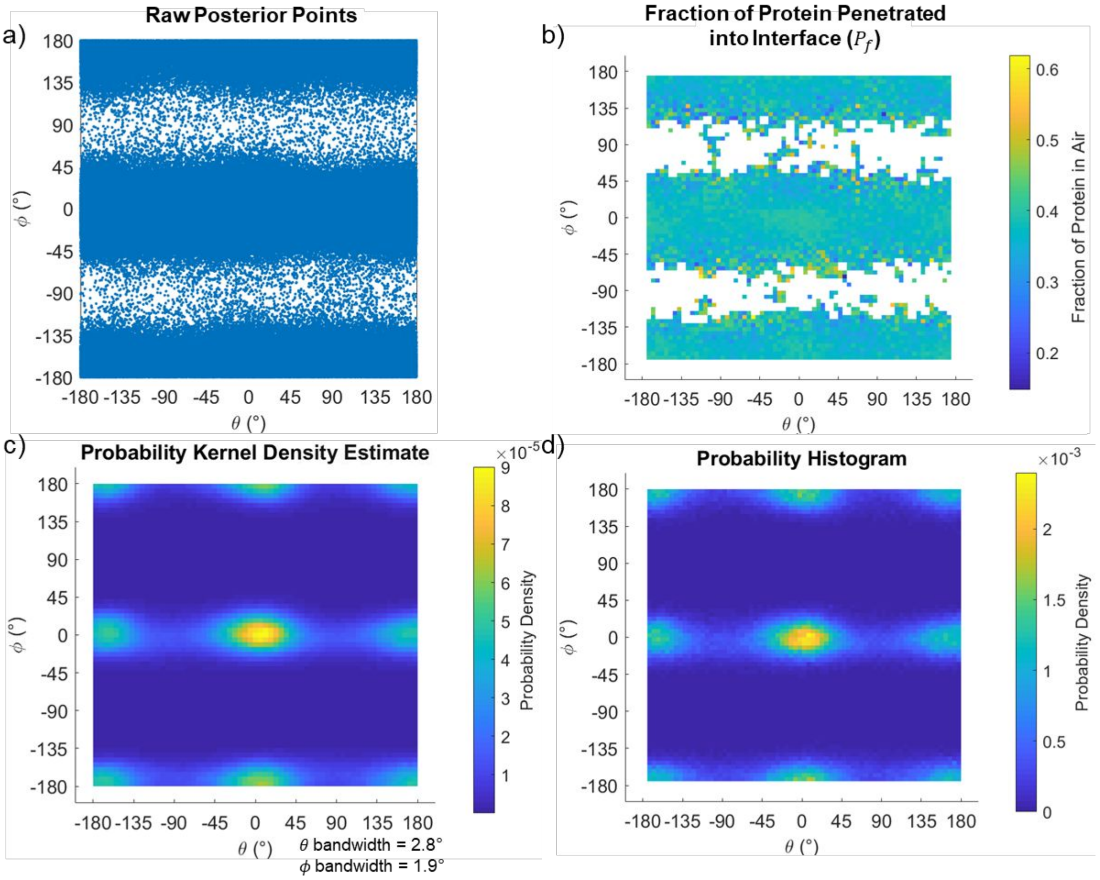

Figure S9. Supporting orientation plots for 50 ppm Fab at the Air/Water interface. As described above, (a) shows the raw posterior points, the θ vs ϕ values for each accepted step in the chain. (b) is a heatmap showing the average fraction of the protein penetrated into the interface, as a function of its rotation based on the accepted steps in the chain, (c) shows the probability density for the orientation space, as determined by Kernel Density Estimate of the accepted steps, while (d) shows the probability density determined by a histogram with 5° by 5° binning.

50 ppm Fab at Oil/Water Interface

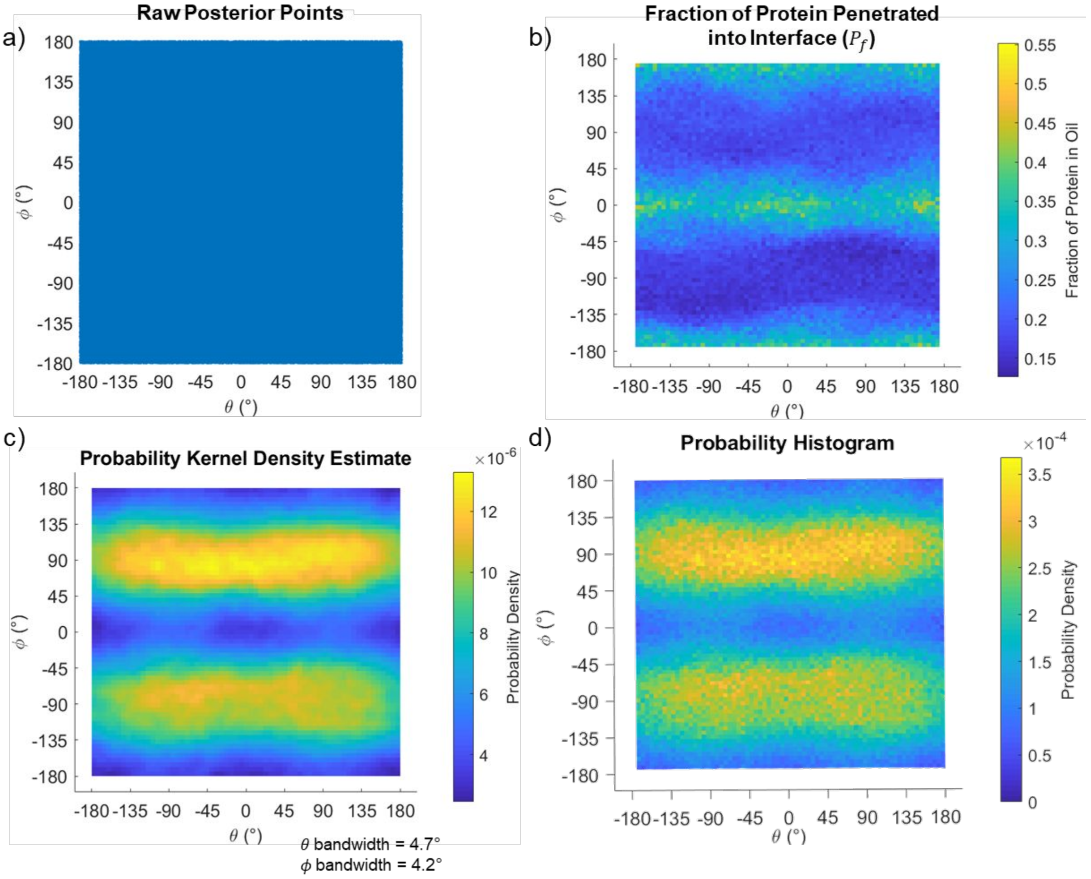

Figure S10. Supporting orientation plots for 50 ppm Fab at the oil/water interface. As described above, (a) shows the raw posterior points, the θ vs ϕ values for each accepted step in the chain. (b) is a heatmap showing the average fraction of the protein penetrated into the interface, as a function of its rotation based on the accepted steps in the chain, (c) shows the probability density for the orientation space, as determined by Kernel Density Estimate of the accepted steps, while (d) shows the probability density determined by a histogram with 5° by 5° binning. For (a) The raw posterior points for 50 ppm Fab at the oil/water interface, the graph appears uniform, because some 2 million points cover the entire plot. This demonstrates that there is only a weak difference in likelihood across the rotation space, and that the measurement is not sensitive enough to accurately determine orientation.

200 ppm Fab at Oil/Water Interface

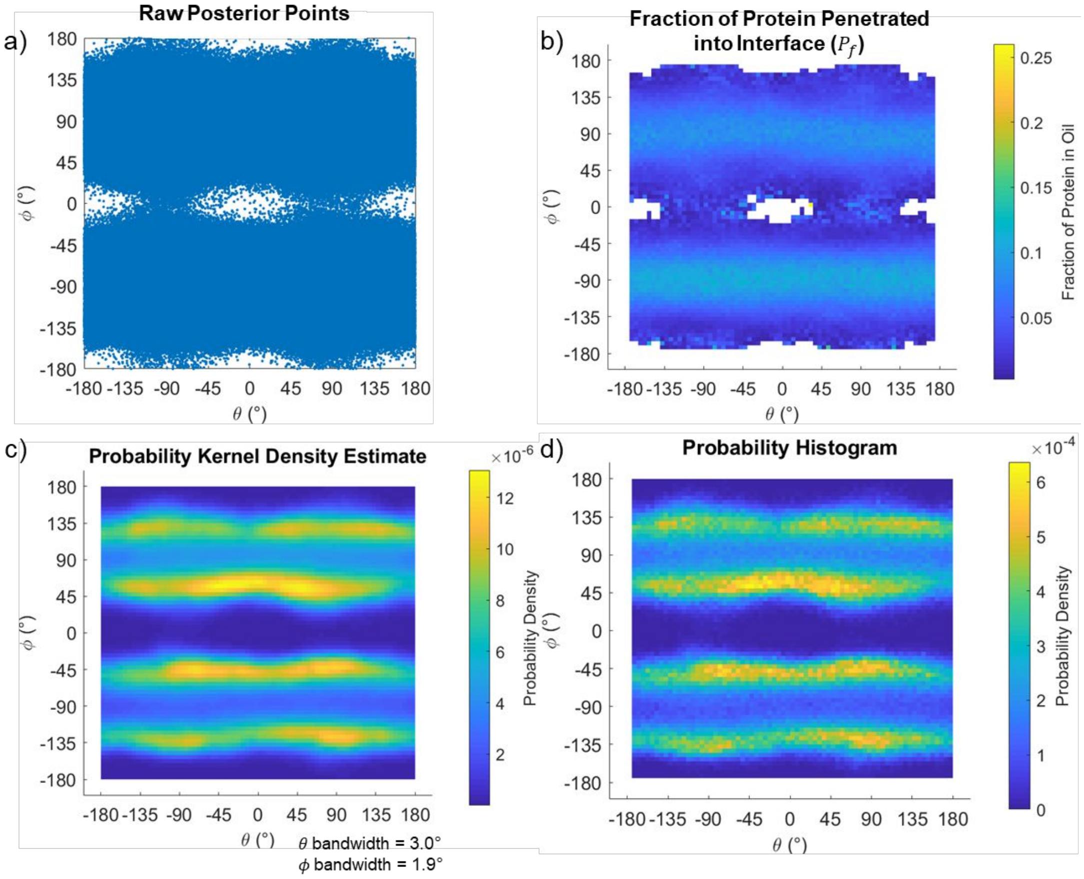

Figure S11. Supporting orientation plots for 200 ppm Fab at the oil/water interface. As described above, (a) shows the raw posterior points, the θ vs ϕ values for each accepted step in the chain. (b) is a heatmap showing the average fraction of the protein penetrated into the interface, as a function of its rotation based on the accepted steps in the chain, (c) shows the probability density for the orientation space, as determined by Kernel Density Estimate of the accepted steps, while (d) shows the probability density determined by a histogram with 5° by 5° binning.

Section 5.2 Fc Fragment

50 ppm Fc at the Air/Water Interface

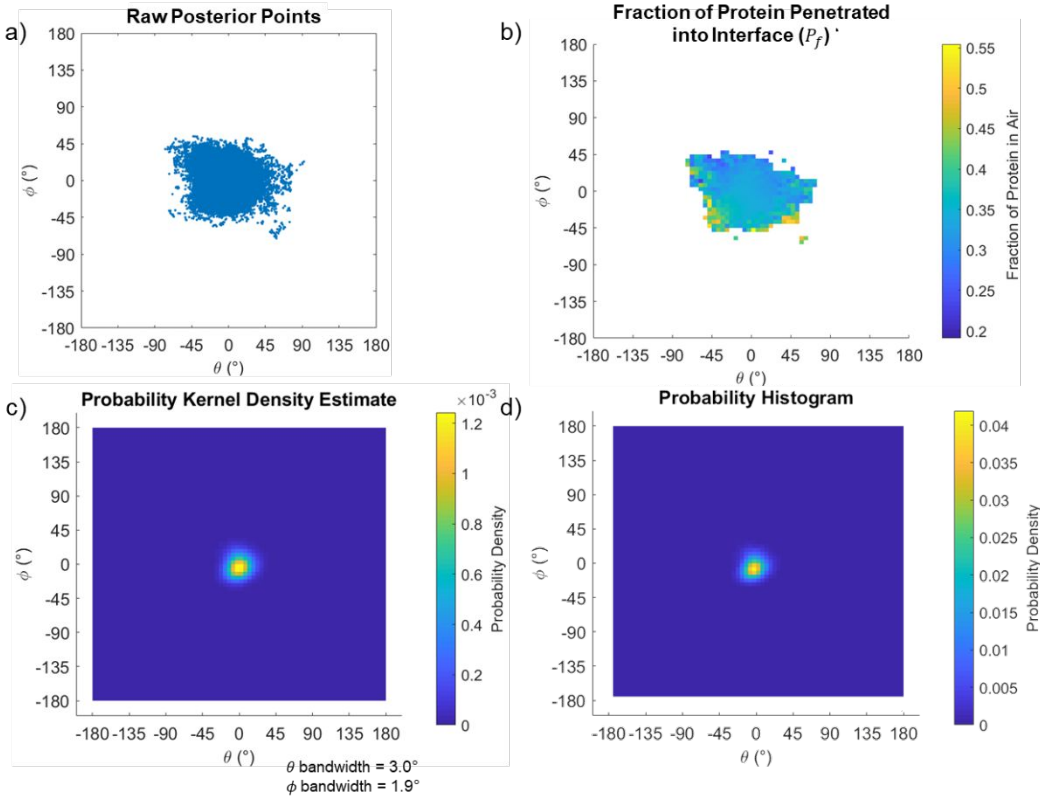

Figure S12. Supporting orientation plots for 50 ppm Fc at the air/water interface. As described above, (a) shows the raw posterior points, the θ vs ϕ values for each accepted step in the chain. (b) is a heatmap showing the average fraction of the protein penetrated into the interface, as a function of its rotation based on the accepted steps in the chain, (c) shows the probability density for the orientation space, as determined by Kernel Density Estimate of the accepted steps, while (d) shows the probability density determined by a histogram with 5° by 5° binning.

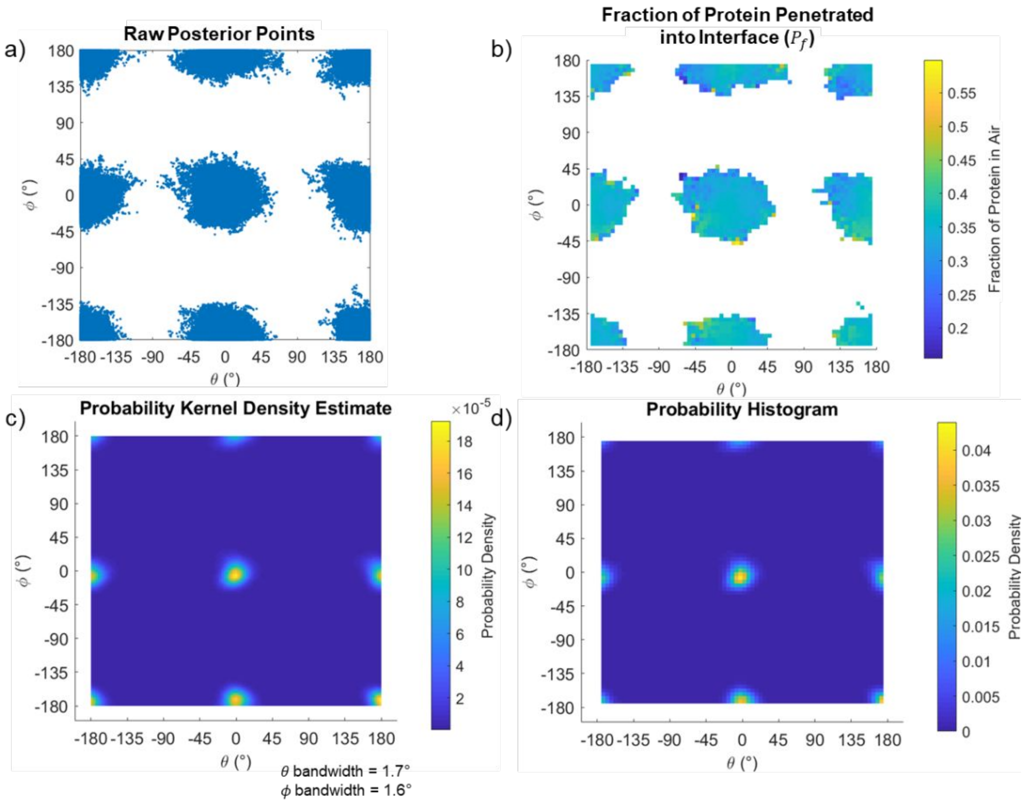

Figure S13. Supporting orientation plots for the combined chain for 50 ppm Fc at the air/water interface. As described above, (a) shows the raw posterior points, the θ vs ϕ values for each accepted step in the chain. (b) is a heatmap showing the average fraction of the protein penetrated into the interface, as a function of its rotation based on the accepted steps in the chain, (c) shows the probability density for the orientation space, as determined by Kernel Density Estimate of the accepted steps, while (d) shows the probability density determined by a histogram with 5° by 5° binning.

.

50 ppm Fc at Oil/Water Interface

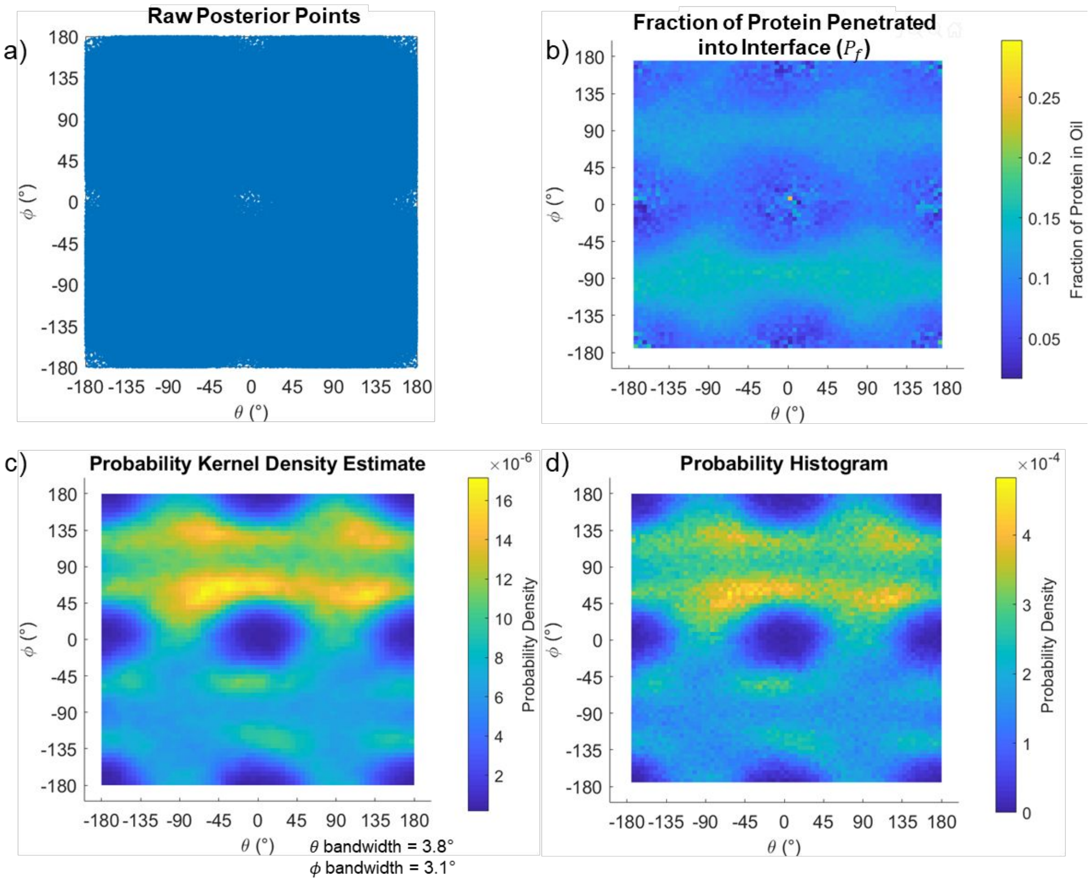

Figure S14. Supporting orientation plots for 50 ppm Fc at the oil/water interface. As described above, (a) shows the raw posterior points, the θ vs ϕ values for each accepted step in the chain. (b) is a heatmap showing the average fraction of the protein penetrated into the interface, as a function of its rotation based on the accepted steps in the chain, (c) shows the probability density for the orientation space, as determined by Kernel Density Estimate of the accepted steps, while (d) shows the probability density determined by a histogram with 5° by 5° binning.

200 ppm Fc at the Oil/Water Interface

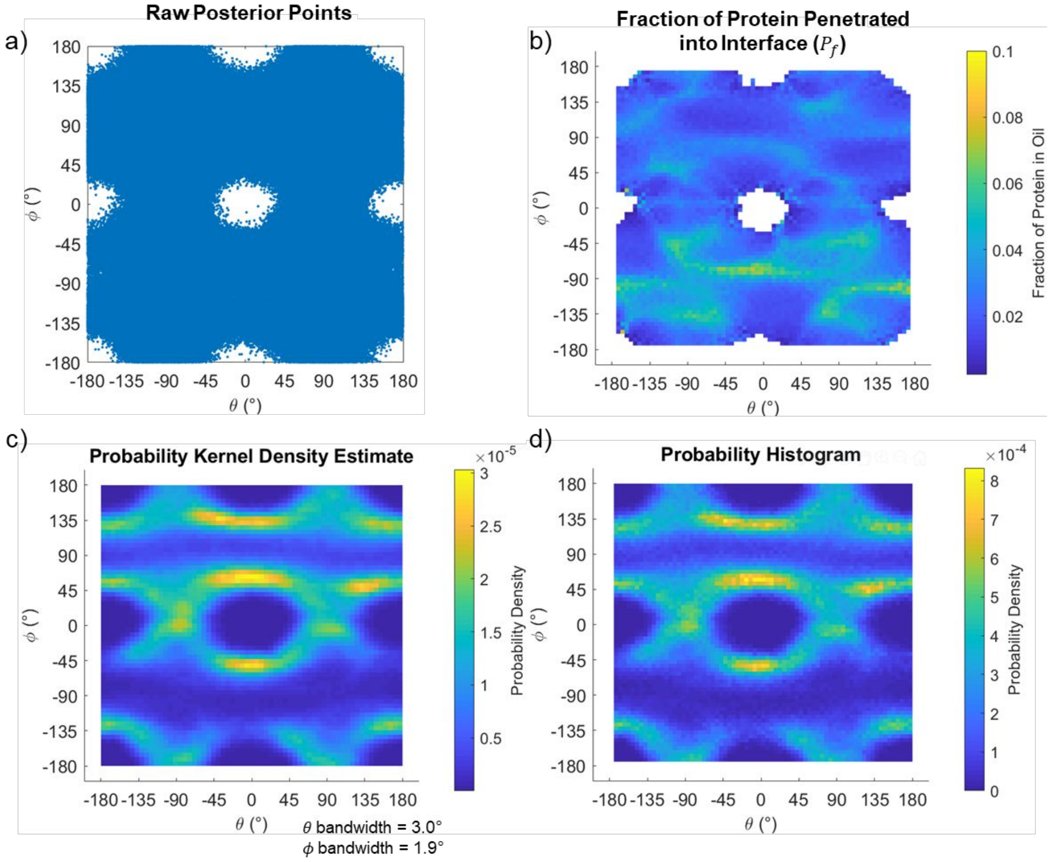

Figure S15. Supporting orientation plots for 200 ppm Fc at the oil/water interface. As described above, (a) shows the raw posterior points, the θ vs ϕ values for each accepted step in the chain. (b) is a heatmap showing the average fraction of the protein penetrated into the interface, as a function of its rotation based on the accepted steps in the chain, (c) shows the probability density for the orientation space, as determined by Kernel Density Estimate of the accepted steps, while (d) shows the probability density determined by a histogram with 5° by 5° binning.

Section 5.3 Full COE-3

50 ppm COE-3 at Air/Water Interface

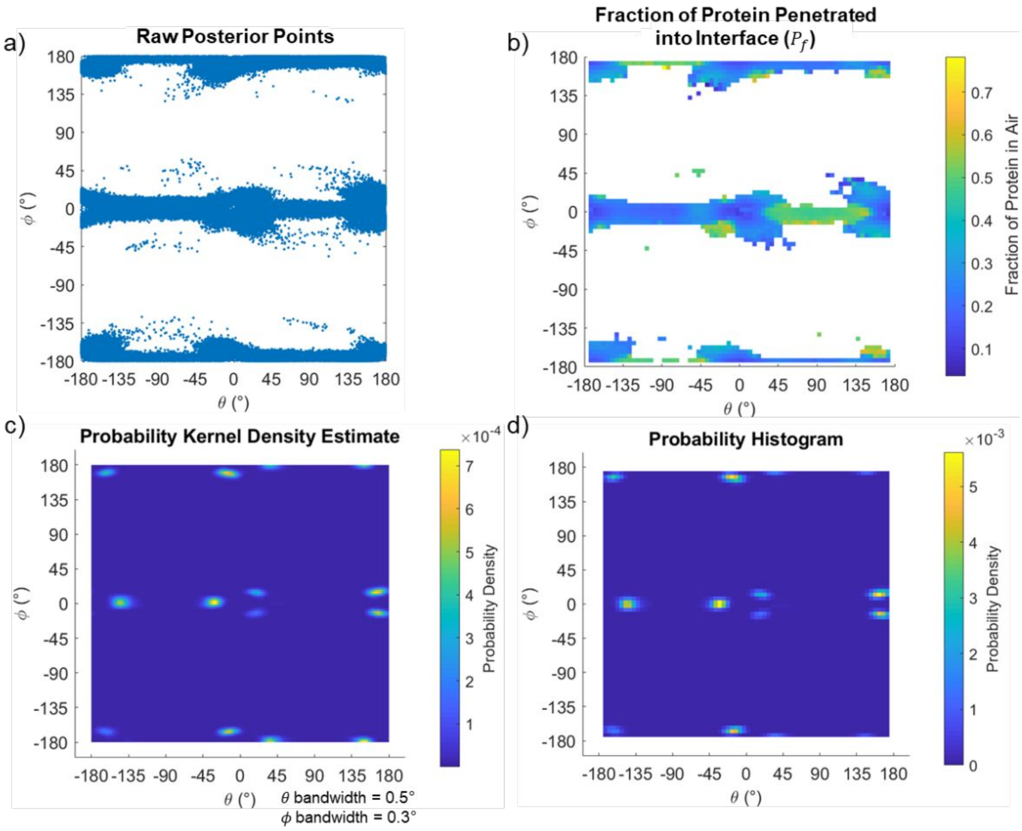

Figure S16. Supporting orientation plots for 50 ppm COE-3 at the Air/Water interface. As described above, (a) shows the raw posterior points, the θ vs ϕ values for each accepted step in the chain. (b) is a heatmap showing the average fraction of the protein penetrated into the interface, as a function of its rotation based on the accepted steps in the chain, (c) shows the probability density for the orientation space, as determined by Kernel Density Estimate of the accepted steps, while (d) shows the probability density determined by a histogram with 5° by 5° binning.

50 ppm COE-3 at Oil/Water Interface

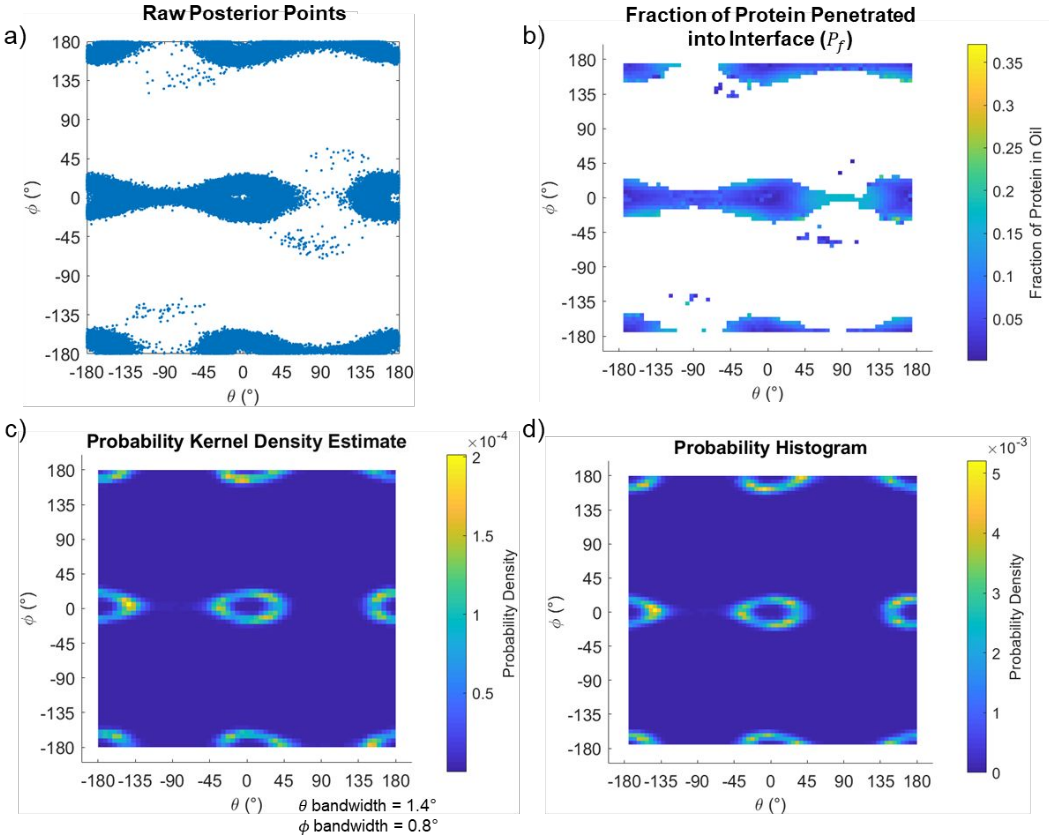

Figure S17. Supporting orientation plots for 50 ppm COE-3 at the oil/water interface. As described above, (a) shows the raw posterior points, the θ vs ϕ values for each accepted step in the chain. (b) is a heatmap showing the average fraction of the protein penetrated into the interface, as a function of its rotation based on the accepted steps in the chain, (c) shows the probability density for the orientation space, as determined by Kernel Density Estimate of the accepted steps, while (d) shows the probability density determined by a histogram with 5° by 5° binning.

200 ppm COE-3 at Oil/Water Interface

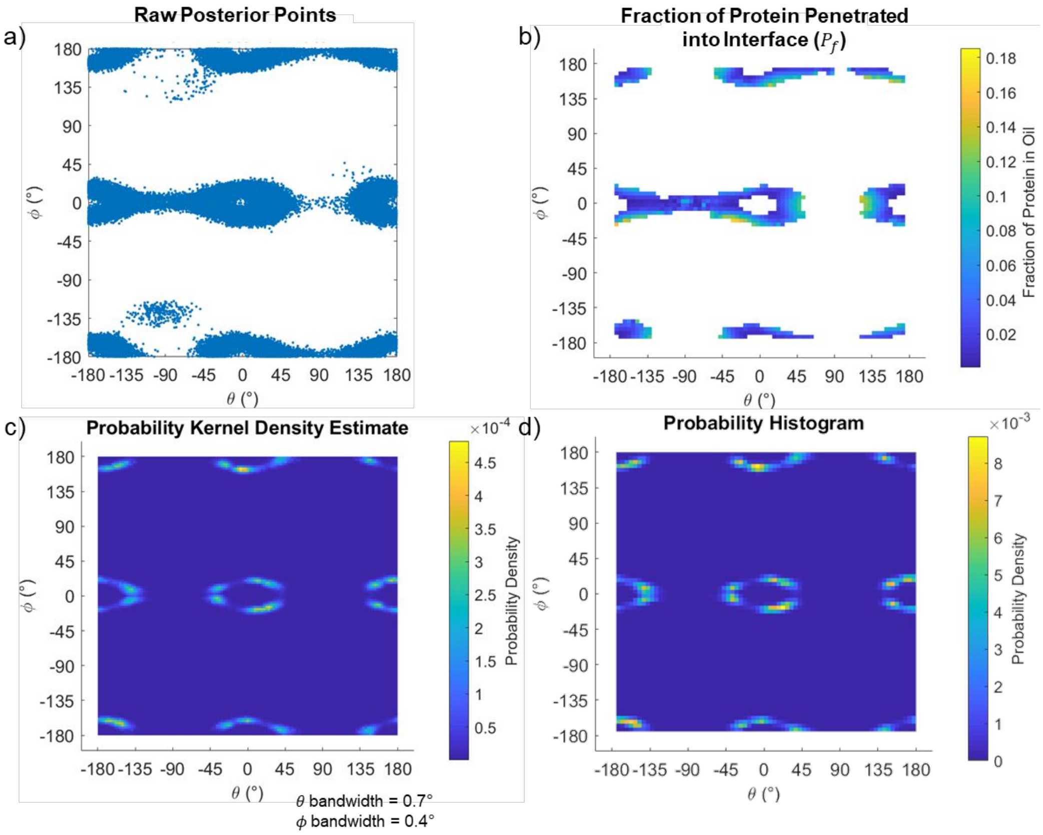

Figure S18. Supporting orientation plots for 200 ppm COE-3 at the oil/water interface. As described above, (a) shows the raw posterior points, the θ vs ϕ values for each accepted step in the chain. (b) is a heatmap showing the average fraction of the protein penetrated into the interface, as a function of its rotation based on the accepted steps in the chain, (c) shows the probability density for the orientation space, as determined by Kernel Density Estimate of the accepted steps, while (d) shows the probability density determined by a histogram with 5° by 5° binning.

References

[1] A.V. Hughes, *RasCAL: Reflectivity Calculations Software*, (2011).

[2] Z. Li, R. Li, C. Smith, F. Pan, M. Campana, J.R.P. Webster, C.F. van der Walle, S. Uddin, S.M. Bishop, R. Narwal, J. Warwicker, J.R. Lu, *Neutron Reflection Study of Surface Adsorption of Fc, Fab, and the Whole mAb*, ACS Appl. Mater. Interfaces. 9 (2017) 23202–23211. https://doi.org/10.1021/acsami.7b06131.

[3] S. Ruane, Z. Li, M. Campana, X. Hu, H. Gong, J.R.P. Webster, F. Uddin, C. Kalonia, S.M. Bishop, C.F. van der Walle, J.R. Lu, *Interfacial Adsorption of a Monoclonal Antibody and Its Fab and Fc Fragments at the Oil/Water Interface*, Langmuir. 35 (2019) 13543–13552. https://doi.org/10.1021/acs.langmuir.9b02317.

[4] C.B. Barber, D.P. Dobkin, H. Huhdanpaa, *The quickhull algorithm for convex hulls*, ACM Trans. Math. Softw. TOMS. 22 (1996) 469–483. https://doi.org/10.1145/235815.235821.

[5] MATLAB, *9.5.0.944444 (R2018b)*, The MathWorks Inc., Natick, Massachusetts, 2018.

[6] B. Guo, J. Menon, B. Willette, *Surface Reconstruction Using Alpha Shapes*, Comput. Graph. Forum. 16 (1997) 177–190. https://doi.org/10.1111/1467-8659.00178.

[7] H.-C. Mahler, F. Senner, K. Maeder, R. Mueller, *Surface Activity of a Monoclonal Antibody*, J. Pharm. Sci. 98 (2009) 4525–4533. https://doi.org/10.1002/jps.21776.

[8] W.A. Link, M.J. Eaton, *On thinning of chains in MCMC*, Methods Ecol. Evol. 3 (2012) 112–115. https://doi.org/10.1111/j.2041-210X.2011.00131.x.
